# A cross-tissue transcriptome-wide association study reveals *GRK4* as a novel susceptibility gene for COPD

**DOI:** 10.1038/s41598-024-80122-w

**Published:** 2024-11-18

**Authors:** Guanglei Chen, Yaxian Jin, Cancan Chu, Yuhao Zheng, Changfu Yang, Yunzhi Chen, Xing Zhu

**Affiliations:** 1 Guizhou University of Traditional Chinese Medicine, Guiyang, 550025 Guizhou China; 2 The First Affiliated Hospital of Guizhou University of Traditional Chinese Medicine, Guiyang, 550000 Guizhou China

**Keywords:** COPD, Cross-tissue TWAS, UTMOST, FUSION, MAGMA, COJO, Diagnostic markers, Predictive markers, Prognostic markers, Respiratory tract diseases, Biomarkers, Genetics research, Molecular medicine, Computational biology and bioinformatics, Gene regulatory networks, Genome informatics, Genetics, Functional genomics, Gene expression, Gene regulation, Genetic association study, Genetic interaction

## Abstract

Chronic obstructive pulmonary disease (COPD) is a prevalent respiratory disorder with environmental factors being the primary risk determinants. However, genetic factors also substantially contribute to the susceptibility and progression of COPD. Although genome-wide association studies (GWAS) have identified several loci associated with COPD susceptibility, the specific pathogenic genes underlying these loci, along with their biological functions and roles within regulatory networks, remain unclear. This lack of clarity constrains our ability to achieve a deeper understanding of the genetic basis of COPD. This study leveraged the FinnGen R11 genetic dataset, comprising 21,617 cases and 372,627 controls, along with GTEx V8 eQTLs data to conduct a cross-tissue transcriptome-wide association study (TWAS). Initially, we performed a cross-tissue TWAS analysis using the Unified Test for Molecular Signatures (UTMOST), followed by validation of the UTMOST findings in single tissues using the Functional Summary-based Imputation (FUSION) method and conditional and joint (COJO) analyses of the identified genes. Subsequently, candidate susceptibility genes were screened using Multi-marker Analysis of Genomic Annotation (MAGMA). The causal relationship between these candidate genes and COPD was further evaluated through summary data-based Mendelian randomization (SMR), colocalization analysis, and Mendelian randomization (MR). Additionally, the identified results were validated against the COPD dataset in the GWAS Catalog (GCST90399694). GeneMANIA was employed to further explore the functional significance of these susceptibility genes. In the cross-tissue TWAS analysis (UTMOST), we identified 17 susceptibility genes associated with COPD. Among these, a novel susceptibility gene, G protein-coupled receptor kinase 4 (GRK4), was validated through single-tissue TWAS (FUSION) and MAGMA analyses, with further confirmation via SMR, MR, and colocalization analyses. Moreover, *GRK4* was validated in an independent dataset. This study identifies *GRK4* as a potential novel susceptibility gene for COPD, which may influence disease risk by exacerbating inflammatory responses. The findings address gaps in previous single-tissue GWAS studies, revealing consistent expression and potential function of *GRK4* across different tissues. However, considering the study’s limitations, further investigation and validation of *GRK4*’s role in COPD are warranted.

**Supplementary Information:**

The online version contains supplementary material available at 10.1038/s41598-024-80122-w.

## Introduction

Chronic obstructive pulmonary disease (COPD) is a progressive and heterogeneous disorder, primarily characterized by persistent airflow limitation, often accompanied by respiratory symptoms such as emphysema and chronic bronchitis, as well as pathological changes including small airway remodeling and abnormal alveolar structures^1^. The predominant symptoms of COPD include dyspnea, chronic cough, and sputum production, frequently associated with lung hyperinflation, which significantly impairs patients’ quality of life^2^. Globally, COPD affects approximately 300 million individuals and is responsible for an estimated 3.2 million deaths annually^3,4^. The disease is marked by a prolonged course, high rates of morbidity and mortality, and substantial treatment costs. This burden is particularly severe in low- and middle-income countries, where COPD imposes increasing economic and psychological stress on individuals and their families, leading to a serious public health challenge^5^. Although smoking, household environment, air pollution, and population aging are recognized as primary risk factors for COPD^6,7^, genetic factors may also heighten the risk by contributing to lung damage and inflammatory responses^8^. However, the pathogenesis of COPD remains incompletely understood, and research on therapeutic strategies is not yet comprehensive. In recent years, the potential of natural compounds and their derivatives in anti-inflammatory, anti-fibrotic, and anti-tumor resistance applications has garnered increasing attention^9–13^. However, the precise therapeutic targets of these compounds remain unknown. Therefore, identifying additional effective drug targets is of significant importance for the prevention and treatment of COPD.

Although diseases such as COPD and asthma are commonly attributed to environmental factors like smoking or exposure to pollutants, research has demonstrated that genetic factors also play a significant role in the risk of developing chronic respiratory diseases^14,15^. The NHGRI-EBI GWAS Catalog has listed 1,150 genetic variants associated with COPD^16^. Additionally, the COPDGene study identified several genetic loci associated with emphysema, gas trapping, and airway traits through computed tomography, including loci near *HHIP*, *15q25*, and *AGER*, as well as novel genetic associations near *SERPINA10* and *DLC1*. The Z variant in *SERPINA1* (*rs28929474T*) exemplifies how GWAS can provide additional insights into COPD risk variants, even though traditional GWAS methods did not identify it as a risk gene^17,18^. However, many of the disease-associated loci identified by GWAS are located in non-coding regions, posing challenges in assessing their functional significance^19^. Moreover, the complexity of linkage disequilibrium (LD) may obscure the identification of causal variants driving these associations^20^.

Compared with traditional GWAS, transcriptome-wide association studies (TWAS) enhance the precision of identifying candidate genes associated with complex traits by integrating expression quantitative trait loci (eQTL) with GWAS summary statistics^20^. This approach not only enables the identification of trait-related genes with greater accuracy but also provides preliminary insights into their regulatory roles, thus addressing GWAS limitations in functional annotation. Although TWAS offers distinct advantages in exploring gene-phenotype associations, conventional single-tissue TWAS methods have notable limitations, particularly in their inability to comprehensively capture the extensive gene expression patterns and regulatory characteristics across multiple tissues in complex, multi-system diseases such as COPD. This constraint may result in the incomplete identification of cross-tissue key genes and their regulatory networks, thereby hindering a comprehensive understanding of the disease’s genetic basis. Consequently, employing cross-tissue TWAS is crucial for investigating the genetic background of multi-system diseases like COPD, as it captures coordinated expression features across tissues, revealing more intricate and systemic pathobiological mechanisms. A more advanced method, known as the Unified Test for Molecular Signatures (UTMOST), extends TWAS by conducting gene-level association analyses across multiple tissues^21^. Unlike single-tissue approaches, UTMOST integrates results from multiple tissues into a unified metric within a summary statistics-based framework, thereby enhancing the accuracy of expression imputation across all available tissues and improving the quantification of overall gene-trait associations. In recent years, cross-tissue association analyses have been widely utilized to identify candidate susceptibility genes for complex multisystem diseases, such as frailty^22^, lung cancer^23^, and ulcerative colitis^24^.

This study aims to identify novel susceptibility genes for COPD through cross-tissue TWAS and validate these findings using multiple independent approaches. Specifically, we integrated COPD GWAS data from FinnGen R11 (discovery set) and the GWAS Catalog (validation set), along with eQTL data from the Genotype-Tissue Expression (GTEx) project V8, to perform cross-tissue TWAS analyses. Subsequently, we evaluated associations within each tissue using Functional Summary-based Imputation (FUSION) and conditional and joint (COJO) analyses^25^, and further validated candidate genes using Multi-marker Analysis of Genomic Annotation (MAGMA)^26^. Additionally, we assessed the causal relationships between candidate genes and COPD through summary-data-based Mendelian Randomization (SMR) and colocalization analyses, as well as Mendelian Randomization (MR). Finally, we explored the biological functions of these genes via GeneMANIA bioinformatics analysis^27^. This multi-layered approach systematically identifies and validates novel pathogenic genes associated with COPD, thereby offering new insights into its underlying pathomechanisms.

## Materials and methods

### Statement on ethical approval

This study utilized publicly available databases, including the GWAS Catalog, FinnGen R11, and GTEx V8, and did not involve any direct experimentation on human subjects or human tissue samples. As such, ethical approval or consent was not required for this research. All analyses were conducted using secondary data derived from these publicly accessible resources.

### Study design

**Fig. 1:**
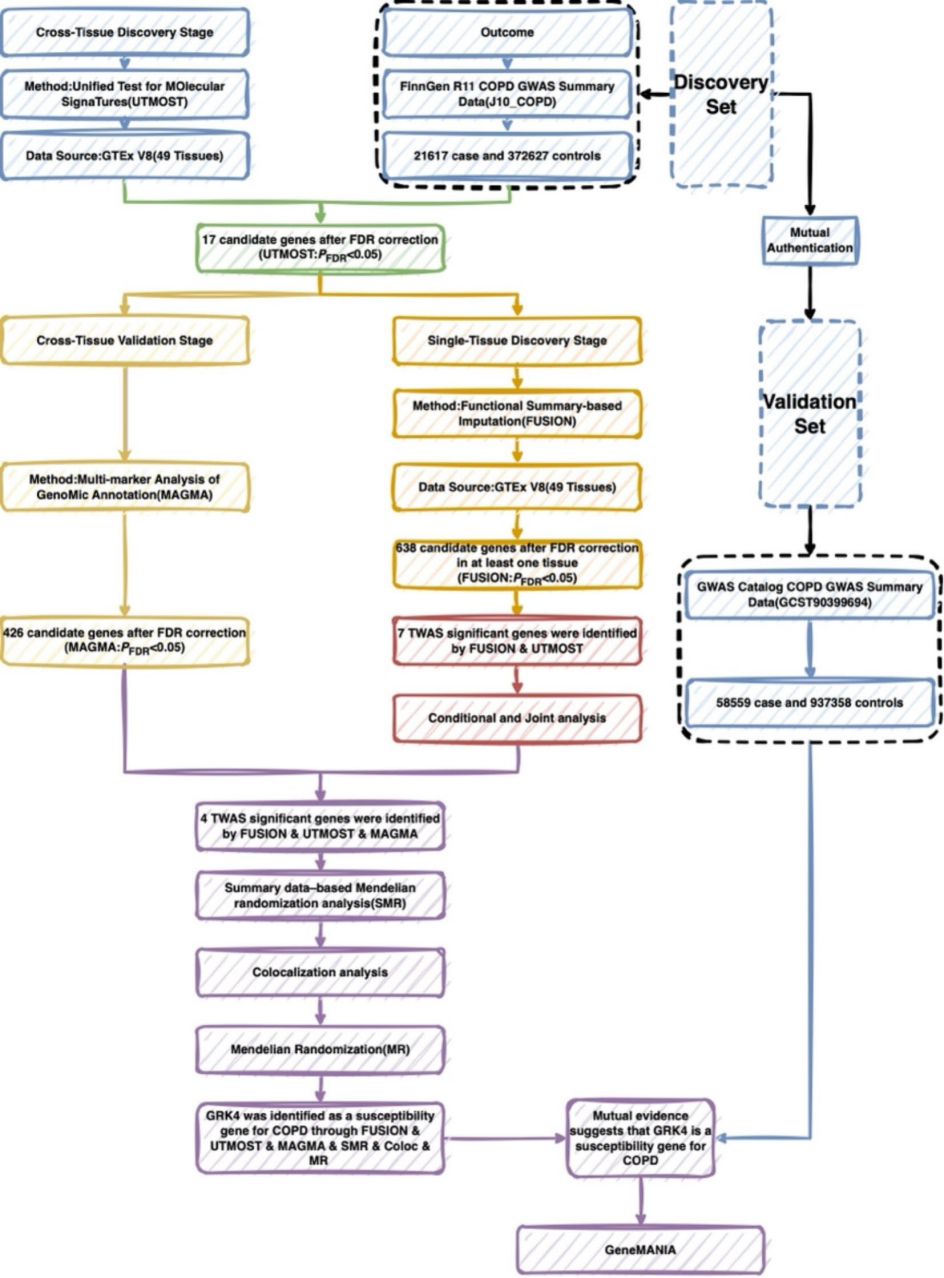
Flowchart of the study design.

The analysis process is shown in Fig. 1.

### COPD data sources

The GWAS data for the COPD discovery cohort were obtained from the FinnGen database, version R11^28^. This database integrates genotypic data from Finnish biobanks with digital health records from the Finnish health registry to explore the relationships between genetic variations and disease trajectories. The FinnGen project aims to expand its study population to 500,000 individuals by the end of 2023, thereby broadening the scope of research. In the FinnGen database, the total sample size for COPD is 394,224, including 21,617 cases and 372,627 controls. Among the 21,617 cases, 5,695 were female, and 15,922 were male. (Data access link: https://r11.risteys.finregistry.fi/endpoints/J10_COPD; Download link: https://storage.googleapis.com/finngen-public-data-r11/summary_stats/finngen_R11_J10_COPD.gz).

The GWAS data for the COPD validation cohort were sourced from the study conducted by Wei Zhou et al.^29^, and are available in the GWAS Catalog database (GCST ID: GCST90399694). This study, conducted under the Global Biobank Meta-analysis Initiative, integrates data across continental biobanks, thereby enhancing the ability to validate diverse GWAS results and improving disease research and risk prediction. The total sample size for COPD in this dataset is 995,917, comprising 58,559 cases and 937,358 controls. (Data access link: https://www.ebi.ac.uk/gwas/studies/GCST90399694; Download link: https://ftp.ebi.ac.uk/pub/databases/gwas/summary_statistics/GCST90399001-GCST90400000/GCST90399694/GCST90399694.tsv.gz).

### eQTL data sources

The eQTL data were derived from GTEx database^30,31^, a large-scale genomics project aimed at investigating how gene expression and genomic variation operate across different human tissues. For this study, we utilized the GTEx V8 dataset, the eighth edition, which includes samples from 49 distinct human tissues. This dataset encompasses gene expression data, genetic variation data, gene co-expression networks, and eQTL analysis results. The GTEx data are widely employed in disease research, gene function studies, and drug development, as they facilitate the understanding of the relationships between genetic variation and disease, elucidate gene expression patterns across various tissues, and support the advancement of personalized medicine.

### UTMOST analysis

In this study, we employed UTMOST to conduct cross-tissue TWAS, a method frequently used to estimate cross-tissue gene expression in TWAS analysis^21^. UTMOST integrates multiple single-tissue association results into a robust metric that quantifies the overall gene-trait association. This approach enhances the ability to identify genes associated with complex traits, thereby overcoming the limitations of single-tissue sample size and increasing the statistical power of the analysis. Subsequently, we applied the Generalized Berk-Jones (GBJ) test to integrate gene-trait associations across single-tissue statistics by accounting for covariance^32^. After adjusting for the false discovery rate (FDR), a significance threshold of FDR < 0.05 was considered statistically significant (UTMOST available at: https://github.com/Joker-Jerome/UTMOST?%20tab=readme-ov-file).

### FUSION analysis

For single-tissue TWAS analysis, we utilized FUSION method, which combines GWAS data for COPD with eQTL data from 49 tissues provided by GTEx V8 to estimate gene-disease associations^33^. We employed genomic data from 1,000 European individuals to estimate LD between single nucleotide polymorphisms (SNPs) at each locus. FUSION integrates multiple statistical models, including BLUP, BSLMM, LASSO, Elastic Net, and Top 1, to evaluate the contribution of each SNP to gene expression. By leveraging different approaches to predict and weight each SNP’s contribution, FUSION provides a comprehensive and accurate assessment^34^. We then combined the genetic effects of COPD (Z-scores from COPD GWAS) with these gene weights to conduct TWAS for COPD (FUSION available at: http://gusevlab.org/projects/fusion/).

### COJO analysis

Following FUSION analysis, it is possible to identify multiple associated traits within a single locus. To determine which of these traits are conditionally independent, we performed COJO analysis, a post-FUSION processing method designed to identify independent genetic signals^33^. COJO analysis accounts for LD between markers, thereby providing a comprehensive understanding of the genetic architecture underlying trait variation. After testing, genes exhibiting independent associations are labeled as “jointly significant,” while those that are no longer significant are considered “marginally significant”^35^.

### MAGMA analysis

We conducted gene analysis using MAGMA software (version 1.08). This tool is utilized for gene-based or gene-set-based association analyses, enabling the identification of functional genes or modules (e.g., gene regulatory pathways) associated with specific traits. MAGMA is also effective in detecting genes associated with multiple low-effect SNPs. In this study, we applied default parameters to aggregate SNP-level association statistics into gene scores, thereby quantifying each gene’s association with the phenotype^36,37^. For detailed information on parameter settings and methodological explanations, please refer to the original MAGMA documentation^26^ (MAGMA available at: https://cncr.nl/research/magma/).

### SMR and bayesian colocalization analysis

We employed SMR to investigate the pleiotropic relationships between gene expression and traits using summary-level data from COPD and eQTL studies. This approach facilitates the identification of COPD causative genes and enhances our understanding of the gene-trait relationship^38–40^. We selected significant SMR probes based on an FDR-corrected SMR *P* < 0.05 and used the HEDI test with a *P* > 0.05 to indicate the absence of heterogeneity^40^ (SMR available at: https://yanglab.westlake.edu.cn/software/smr/#SMR&HEIDIanalysis).

Subsequently, we performed Bayesian colocalization analysis using the “coloc” R package (version 5.2.3) to determine whether GWAS and eQTL signals overlap at causal variant sites^41,42^. This analysis computes posterior probabilities for five hypotheses (PPH): (1) no association with either trait (H_0_), (2) association with only trait 1 (H_1_), (3) association with only trait 2 (H_2_), (4) association with both traits due to different causal variants (H_3_), and (5) association with both traits due to the same causal variant (H_4_). Following the literature, we defined colocalization when PPH_4_ > 0.8 and moderate colocalization when PPH_4_ > 0.5^43^ (“coloc” R package available at: https://github.com/chr1swallace/coloc).

### MR analysis

We conducted MR analysis using the “TwoSampleMR” R package (version 0.6.6). In this analysis, we used cis-eQTL SNPs as instrumental variables (IVs), gene expression as the exposure, and COPD GWAS data as the outcome. IVs were required to have an *R*^*2*^ < 0.001 and LD = 10,000 kb. Since only one independent IV was available, we employed the Wald ratio as the primary method for estimating MR effects, with a significance threshold set at *P* < 0.05^44^ (“TwoSampleMR” R package available at: https://github.com/MRCIEU/TwoSampleMR?tab=readme-ov-file).

### GeneMANIA analysis

Finally, we used GeneMANIA to analyze the complex relationships and biological functions among genes. The GeneMANIA platform integrates various datasets on genetic interactions, pathways, and co-expression of target genes^27^ (GeneMANIA platform available at: https://genemania.org/).

## Results

### Cross-tissue and single-tissue TWAS analysis

In the discovery set for COPD, cross-tissue TWAS analysis identified a total of 352 genes with *P* < 0.05 (Supplementary Material 1, S1), of which 17 genes remained significant after stringent FDR correction (*P*_*FDR*_ < 0.05) (Table 1). These significant genes suggest potential roles in the pathophysiological processes of COPD and provide candidate targets for further investigation. To enhance the rigor and reliability of our analysis, we conducted validation through single-tissue TWAS, identifying a total of 638 genes that remained significant post-FDR correction (*P*_*FDR*_ < 0.05) in at least one tissue (Supplementary Material 1, S2), potentially reflecting tissue-specific regulatory mechanisms involved in COPD.

**Fig. 2:**
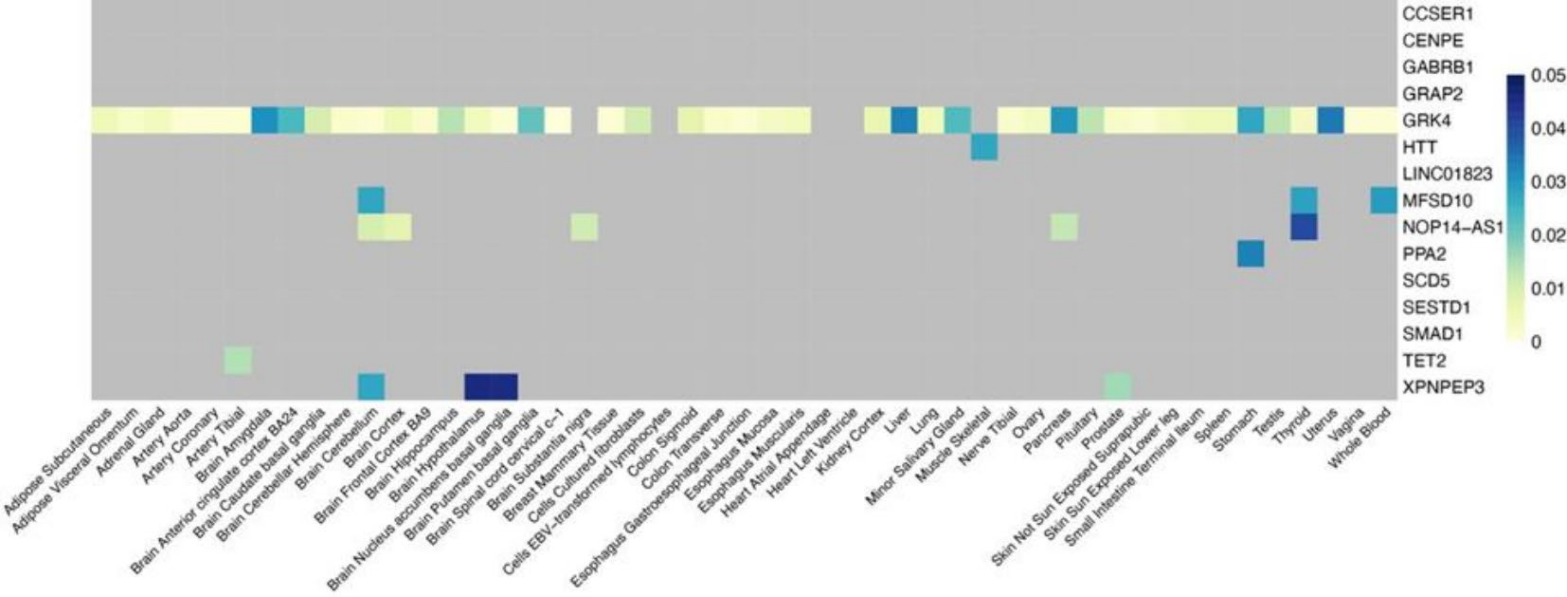
Heatmap (Note: The heatmap of 17 genes identified by UTMOST is separated by tissue in FUSION; gray boxes indicate *P*_*FDR*_ > 0.05 in FUSION.). Note: The heatmap was generated using R (version 4.4.0) with the R packages: pheatmap (version 1.0.12) and reshape2 (version 1.4.4).

Further analysis revealed that the 17 genes identified through cross-tissue TWAS also demonstrated consistent significance in single-tissue TWAS, as detailed in Fig. 2. This concordance not only reinforces the findings from the cross-tissue analysis but also highlights the broad expression and potential functions of these genes across different tissues. Among the genes achieving strict significance thresholds in both cross-tissue and single-tissue analyses, seven candidates were identified, comprising six protein-coding genes (*MFSD10*, *GRK4*, *PPA2*, *TET2*, *XPNPEP3*, and *HTT*) and one long non-coding RNA (lncRNA) gene (*NOP14-AS1*) (Supplementary Material 1, S3). The identification of these genes provides valuable insights into the molecular mechanisms underlying COPD, particularly regarding the roles of protein-coding and non-coding RNAs in disease pathology.

**Table 1:**
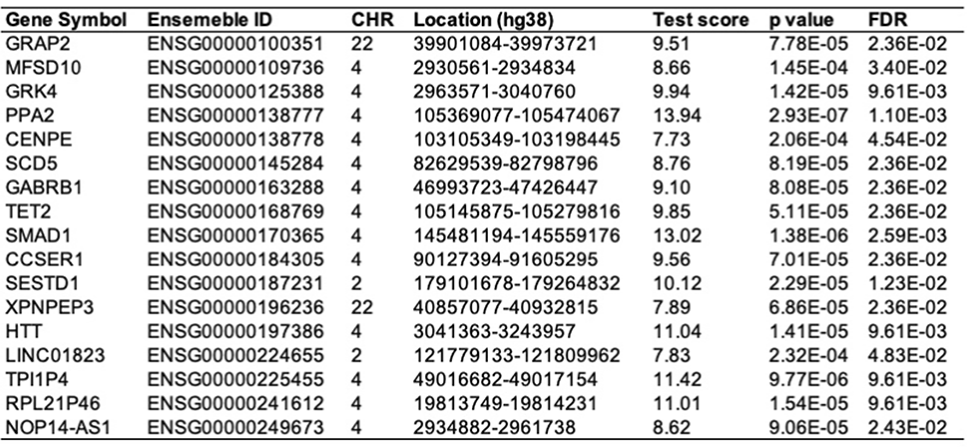
Key genes associated with COPD risk identified in the cross-tissue UTMOST analysis.

### COJO analysis

In the COPD discovery set, seven candidate genes primarily located on chromosomes 4 and 22 (*MFSD10*, *GRK4*, *PPA2*, *TET2*, *XPNPEP3*, *HTT*, and *NOP14-AS1*) were further analyzed using COJO analysis within each tissue to validate their associations with COPD and eliminate potential false positives due to LD. The results indicated that *GRK4* remained significantly associated independently of other genes, suggesting its possible key role in COPD pathogenesis (Supplementary Material 1, S4 and Supplementary Material 2).

Notably, while *PPA2*, *TET2*, and *HTT* exhibited significance in single-tissue TWAS analyses, the presence of LD may have confounded these results. Consequently, these genes did not meet the stringent criteria for multi-layer validation and were excluded from subsequent in-depth analysis. This selection process underscores the rigorous control strategies employed in this study to ensure the reliability of candidate genes and the accuracy of our findings. By mitigating potential false positives, these analyses offer more precise genetic targets for COPD molecular studies and lay a robust foundation for future investigations.

### MAGMA analysis

**Fig. 3:**
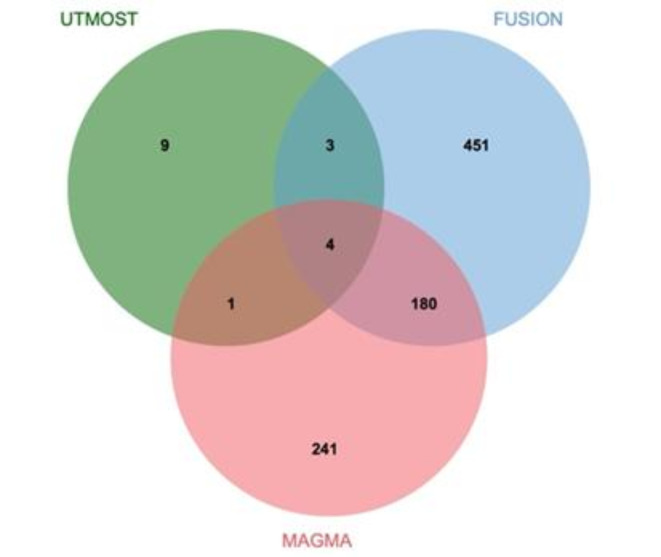
Venn Diagram (Note: MAGMA identified 426 significant genes associated with COPD, FUSION identified 638, and UTMOST cross-tissue analysis identified 17. Among these, four genes—*MFSD10*, *GRK4*, *TET2*, and *HTT*—were commonly identified across all analyses.).

In the COPD discovery set, MAGMA analysis identified a total of 426 genes significantly associated with COPD (*P*_*FDR*_ < 0.05) (Supplementary Material 1, S5). These genes may play crucial roles in COPD pathogenesis, providing numerous candidate targets for further exploration of COPD molecular mechanisms. To enhance the robustness and consistency of our findings, we performed an integrative analysis combining the UTMOST cross-tissue analysis results with key genes detected by FUSION and MAGMA, aiming to identify core genes highly relevant to COPD. This integrative approach ultimately confirmed four critical candidate genes: *MFSD10*, *GRK4*, *TET2*, and *HTT* (Fig. 3). These genes demonstrated significance across multiple analytical methods, suggesting consistent genetic associations and potential biological functions under varied tissues and conditions.

### SMR and colocalization analysis

SMR analysis provides estimates of causal effects of genetic variation on phenotypes, while colocalization analysis further assesses whether these causal associations share the same genetic signals between gene expression and phenotype, thereby enhancing the credibility and rigor of causal inference. In the COPD discovery set, we selected four key genes—*MFSD10*, *GRK4*, *TET2*, and *HTT*—for SMR and colocalization analyses within their respective tissues to strengthen the robustness of the results. SMR analysis revealed that *MFSD10*, *TET2*, and *HTT* did not exhibit significant causal effects on COPD in their corresponding tissues (*P*_*SMR−FDR*_ > 0.05), nor did they share the same genetic signals with COPD in colocalization analysis (PP.H4 < 50%), suggesting that changes in their expression may not directly contribute to COPD pathogenesis (Supplementary Material 1, S6 and S7).

**Fig. 4:**
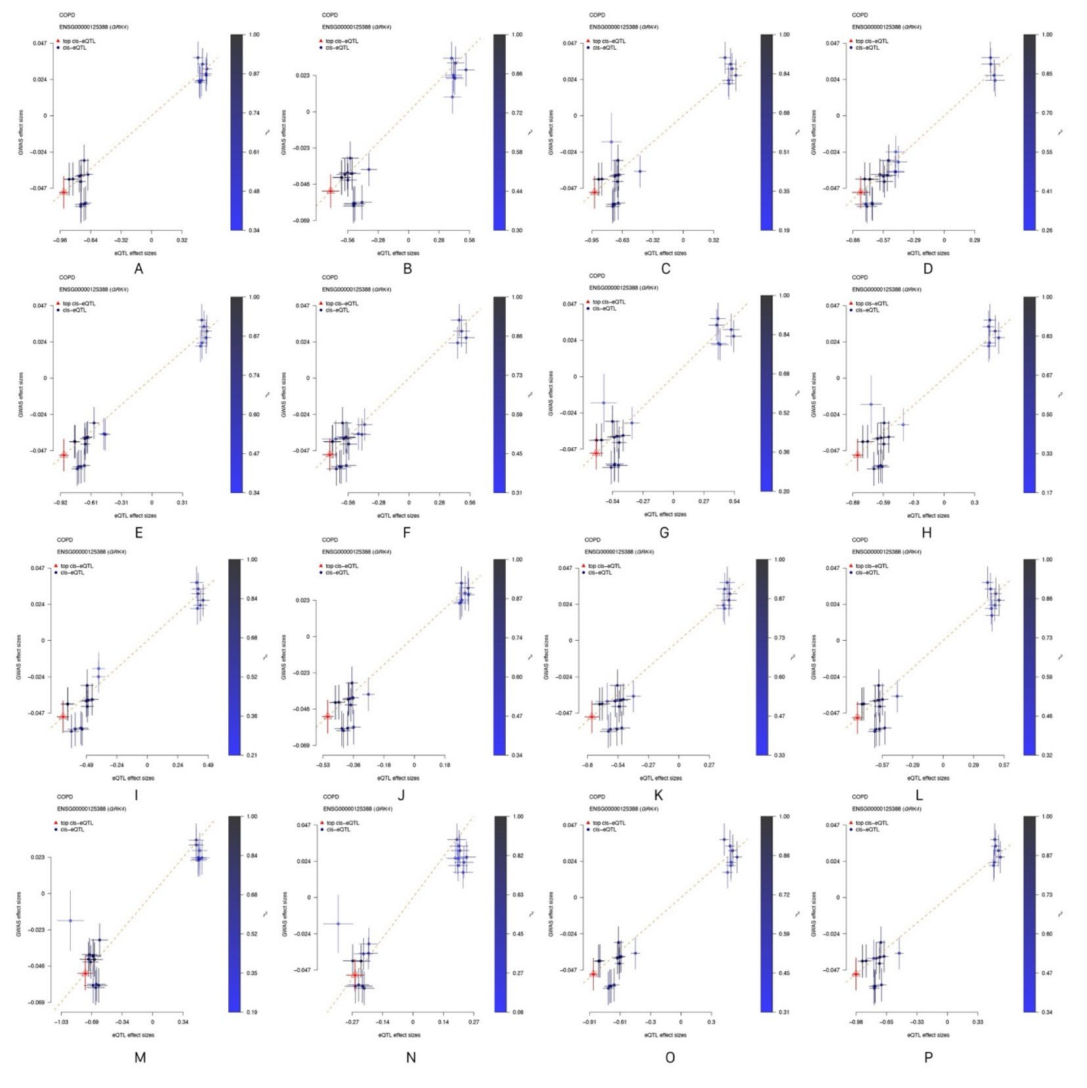
Effect plots from SMR analysis. (Note: A: in Adipose Visceral Omentum; B: in Adrenal Gland; C: in Artery Aorta; D: in Artery Coronary; E: in Artery Tibial; F: in Brain Cerebellar Hemisphere; G: in Brain Cerebellum; H: in Breast Mammary Tissue; I: in Colon Sigmoid; J: in Colon Transverse; K: in Esophagus Gastroesophageal Junction; L: in Esophagus Muscularis; M: in Ovary; N: in Pituitary; O: in Skin Sun Exposed Lower leg; P: in Thyroid).

**Fig. 5:**
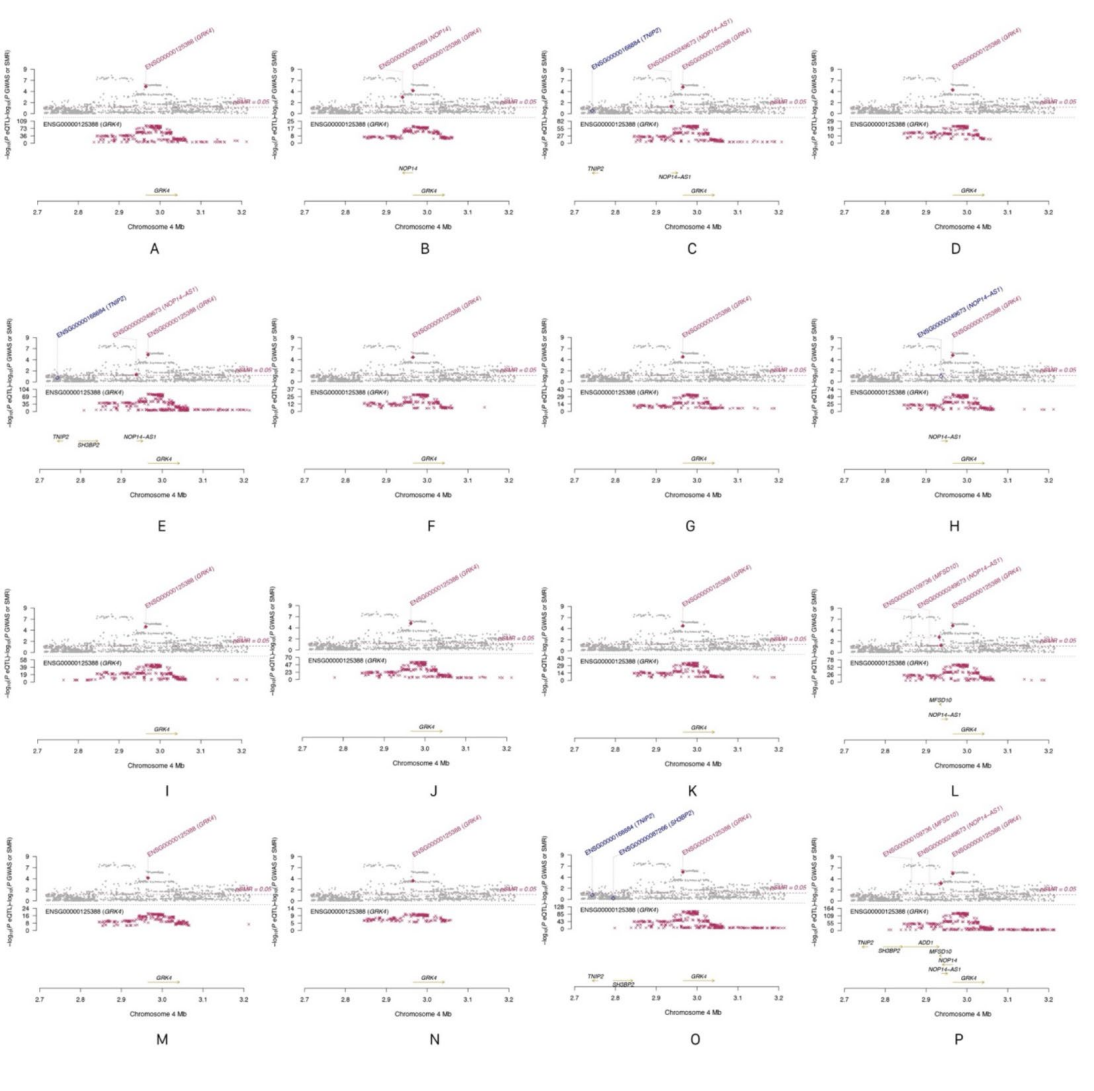
Locus plots from the SMR analysis. (Note: A: in Adipose Visceral Omentum; B: in Adrenal Gland; C: in Artery Aorta; D: in Artery Coronary; E: in Artery Tibial; F: in Brain Cerebellar Hemisphere; G: in Brain Cerebellum; H: in Breast Mammary Tissue; I: in Colon Sigmoid; J: in Colon Transverse; K: in Esophagus Gastroesophageal Junction; L: in Esophagus Muscularis; M: in Ovary; N: in Pituitary; O: in Skin Sun Exposed Lower leg; P: in Thyroid).

**Fig. 6:**
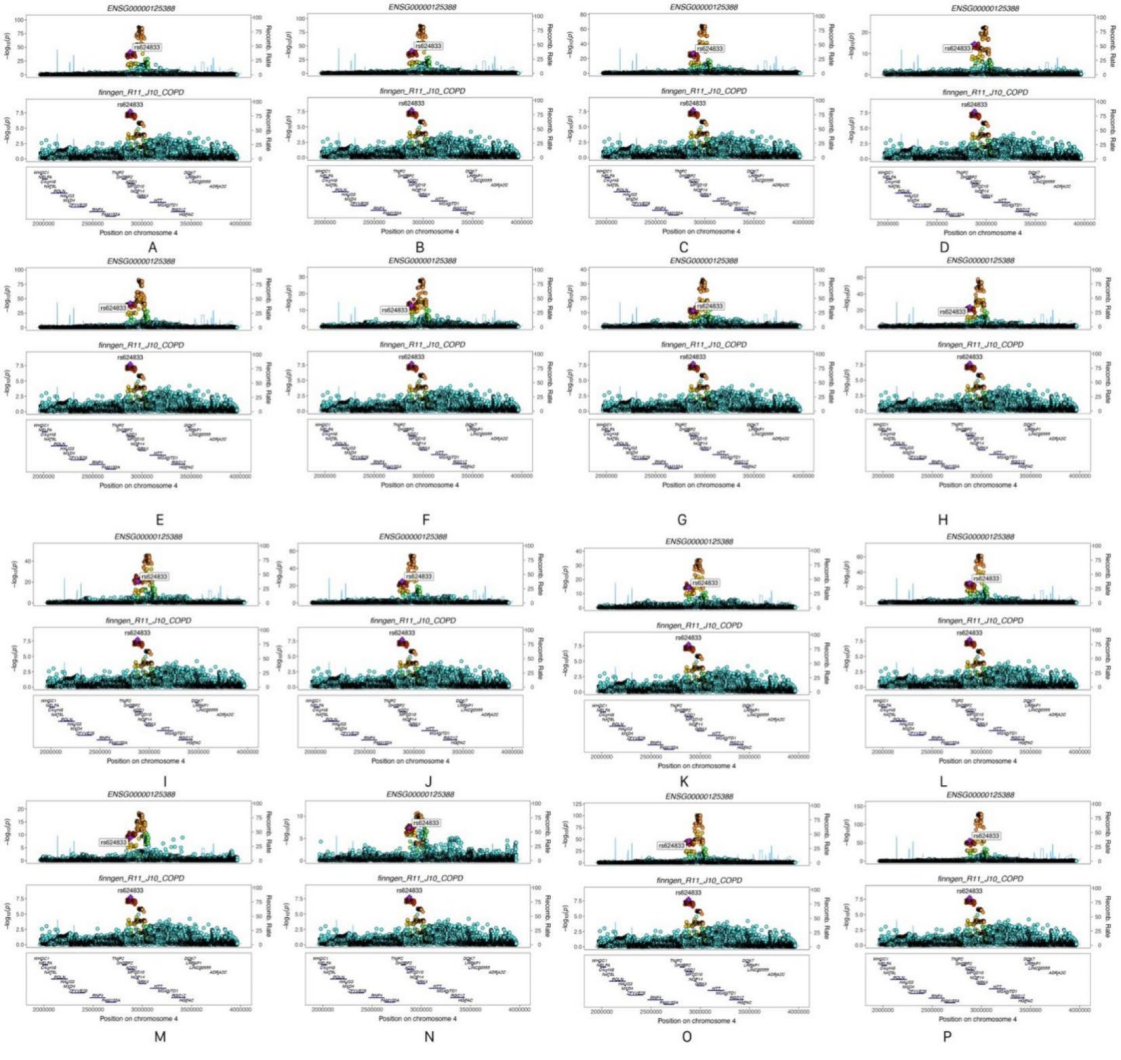
Colocalization analysis plots. (Note: A: in Adipose Visceral Omentum; B: in Adrenal Gland; C: in Artery Aorta; D: in Artery Coronary; E: in Artery Tibial; F: in Brain Cerebellar Hemisphere; G: in Brain Cerebellum; H: in Breast Mammary Tissue; I: in Colon Sigmoid; J: in Colon Transverse; K: in Esophagus Gastroesophageal Junction; L: in Esophagus Muscularis; M: in Ovary; N: in Pituitary; O: in Skin Sun Exposed Lower leg; P: in Thyroid).

In contrast, *GRK4* demonstrated significant causal effects on COPD across 16 different tissues, supporting its potential biological role in COPD. Moreover, colocalization analysis indicated that *GRK4* shares the same genetic signal with COPD, specifically SNP *rs624833* (moderate colocalization, PP.H4 > 50%). This finding suggests that *GRK4* may play a crucial role in the pathological mechanisms of COPD. Detailed data are presented in Table 2; Figs. 4 and 5, and 6. The integration of SMR and colocalization analyses thus further substantiates the significance of *GRK4*, providing strong support for its potential as a therapeutic target for COPD.

**Table 2:**
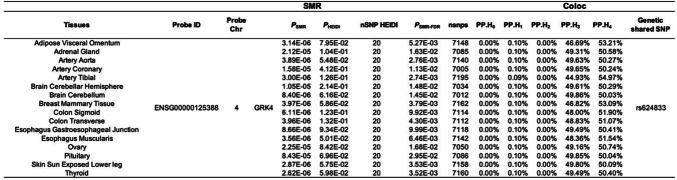
SMR and colocalization analysis results for *GRK4*. **Tissue**: Tissue types analyzed; **Probe ID**: Gene probe identifier used for analysis; **Probe Chr**: Chromosome number where the gene is located; ***P***_**SMR**_: p-value for the SMR analysis; ***P***_**HEIDI**_: p-value for the HEIDI test, used to assess the heterogeneity of the SNPs; **nSNP HEIDI**: Number of SNPs included in the HEIDI test; ***P***_**SMR−FDR**_: FDR adjusted p-value for the SMR analysis; **nsnps**: Total number of SNPs included in the analysis; **PP.H**_**0**_, **PP.H**_**1**_, **PP.H**_**2**_, **PP.H**_**3**_, **PP.H**_**4**_: Posterior probabilities for different hypotheses in the colocalization analysis; Specific SNP indicating significant genetic sharing between the trait and the gene.

### MR analysis

**Fig. 7:**
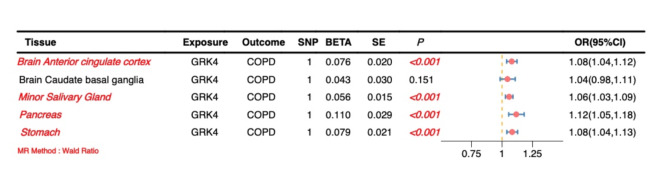
Forest plot of the causal effect of *GRK4* on COPD.

MR analysis demonstrated significant positive causal effects of *GRK4* on COPD across multiple tissues, further supporting its potential pathogenic role in the disease. Specifically, significant positive causal associations between *GRK4* and COPD were observed in the brain anterior cingulate cortex, minor salivary gland, pancreas, and stomach (*P* < 0.05, OR > 1), indicating that upregulation of *GRK4* expression in these tissues may increase the risk of COPD. These findings suggest that *GRK4* may influence COPD development through multiple pathways across different tissues (Fig. 7, Supplementary Material 1, S8).

Notably, MR analysis did not show a significant causal effect of *GRK4* on COPD in the brain caudate basal ganglia (*P* > 0.05), which may reflect tissue-specific effects of *GRK4* or differential mechanisms in COPD pathogenesis across tissues. This observation underscores the need for further investigation to elucidate the functional differences of *GRK4* in various tissues and physiological contexts.

### Validation of *GRK4* efficacy

To further validate the role of the *GRK4* gene in COPD, we conducted a cross-tissue TWAS analysis in the COPD validation set, identifying 301 candidate genes with *P* < 0.05 (Supplementary Material 1, S9), among which 27 genes remained significant after FDR correction (*P*_*FDR*_ < 0.05), as detailed in Table 3. Additionally, for single-tissue TWAS validation, 980 significant genes (*P*_*FDR*_ < 0.05) were identified in at least one tissue (Supplementary Material 1, S10). Cross-validation between cross-tissue and single-tissue analyses revealed 11 candidate genes meeting stringent significance thresholds, including nine protein-coding genes (*GPD2*, *GRK4*, *HHIP*, *IL18R1*, *MAP4K4*, *MFSD10*, *MSANTD1*, *SLC9A2*, and *TCF4*) and two lncRNAs (*NOP14-AS1* and *TEX41*) (Table 4, Supplementary Material 1, S11).

**Table 3:**
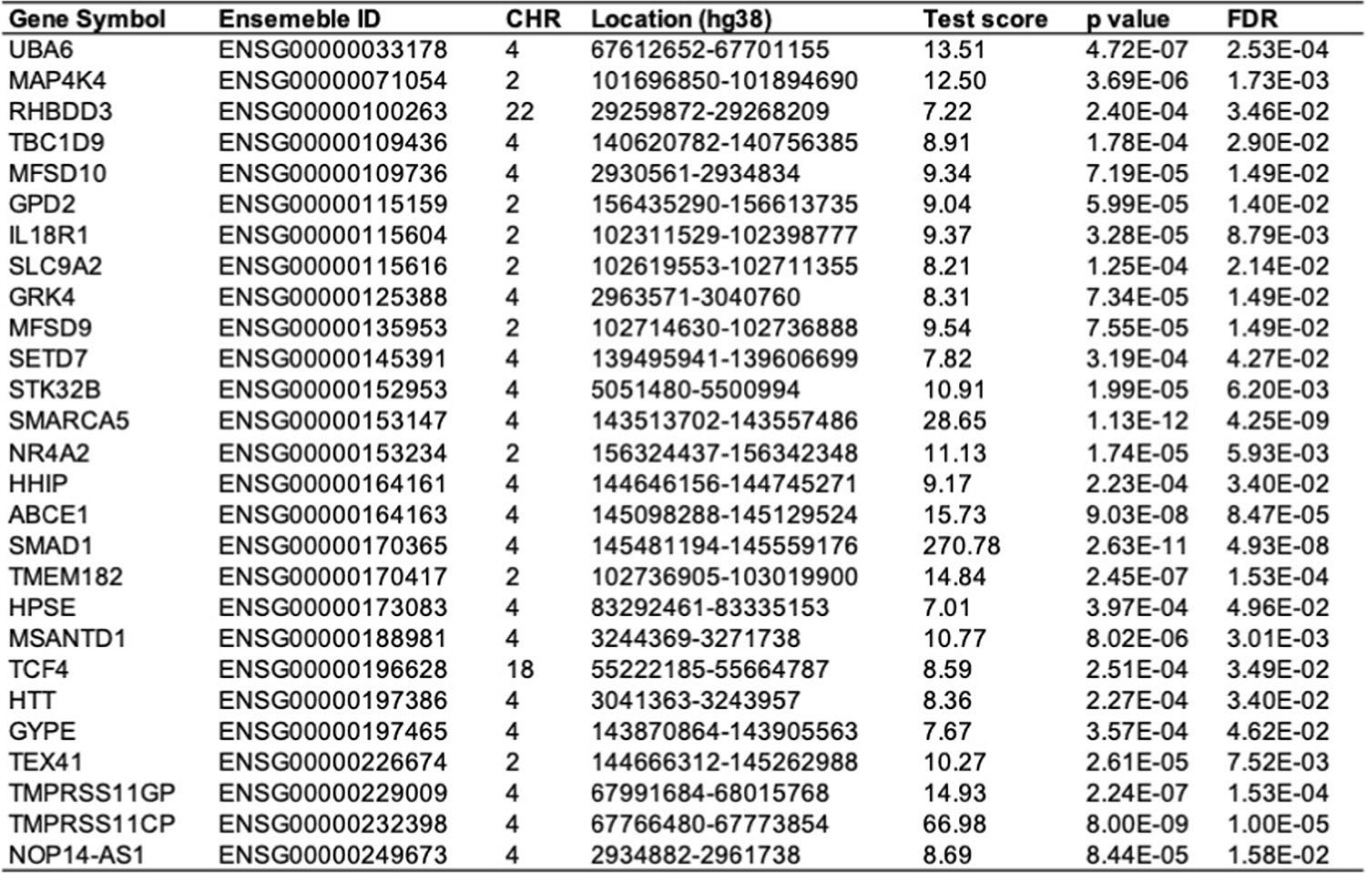
Key genes associated with COPD risk identified in the cross-tissue UTMOST analysis.

**Table 4:**
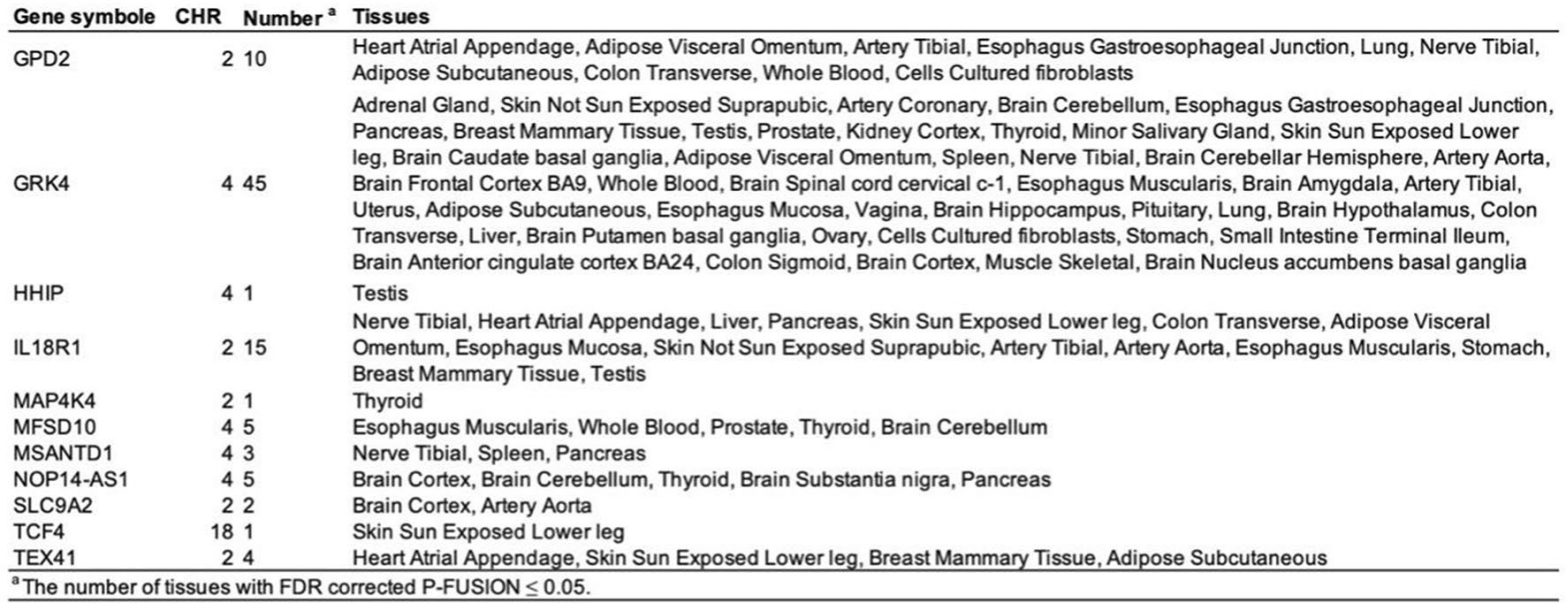
Eleven significant genes identified in the FUSION analysis from the cross-tissue UTMOST study. **Gene symbol**: The symbol representing each gene. ;**CHR**: The chromosome number where each gene is located. ;**Number**: The number of tissues in which each gene showed significance with FDR-corrected *P*_FUSION_ < 0.05.; **Tissues**: The specific tissues where significant associations were identified for each gene.

**Fig. 8:**
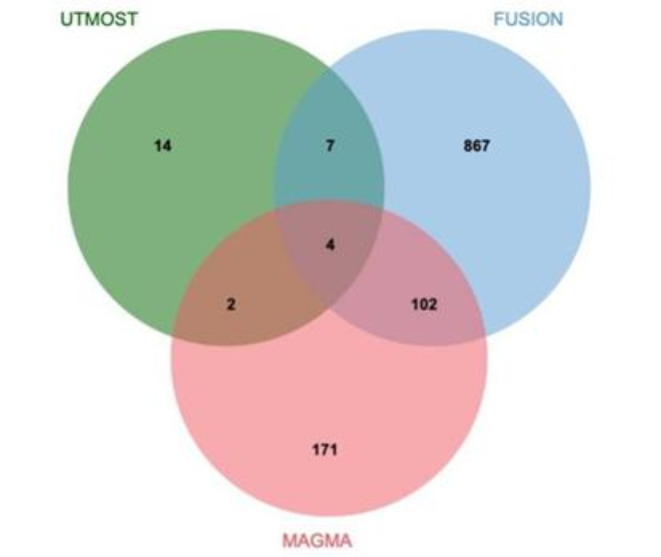
Venn diagram (Note: MAGMA identified 279 key genes associated with COPD, FUSION identified 980, and UTMOST cross-tissue analysis identified 27, of which 4 are common: *MAP4K4*, *GRK4*, *MSANTD1*, and *TCF4*).

To eliminate potential false-positive results due to LD for the 11 candidate genes located on chromosomes 2, 4, 18, and 22, we performed COJO analysis in their respective tissues (Supplementary Material 1, S12). Additionally, MAGMA analysis confirmed 279 genes significantly associated with COPD (*P*_*FDR*_ < 0.05) (Supplementary Material 1, S13). To enhance the reliability of our results, we integrated the UTMOST cross-tissue analysis findings with significant genes detected by FUSION and MAGMA, ultimately identifying four key candidate genes: *MAP4K4*, *GRK4*, *MSANTD1*, and *TCF4* (Table 5; Fig. 8).

**Fig. 9:**
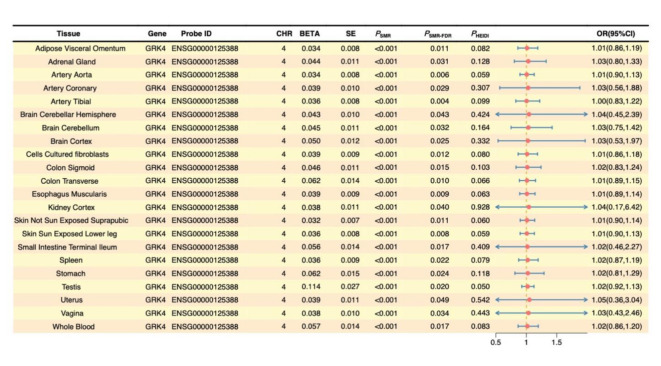
Forest plot of SMR analysis.

**Fig. 10:**
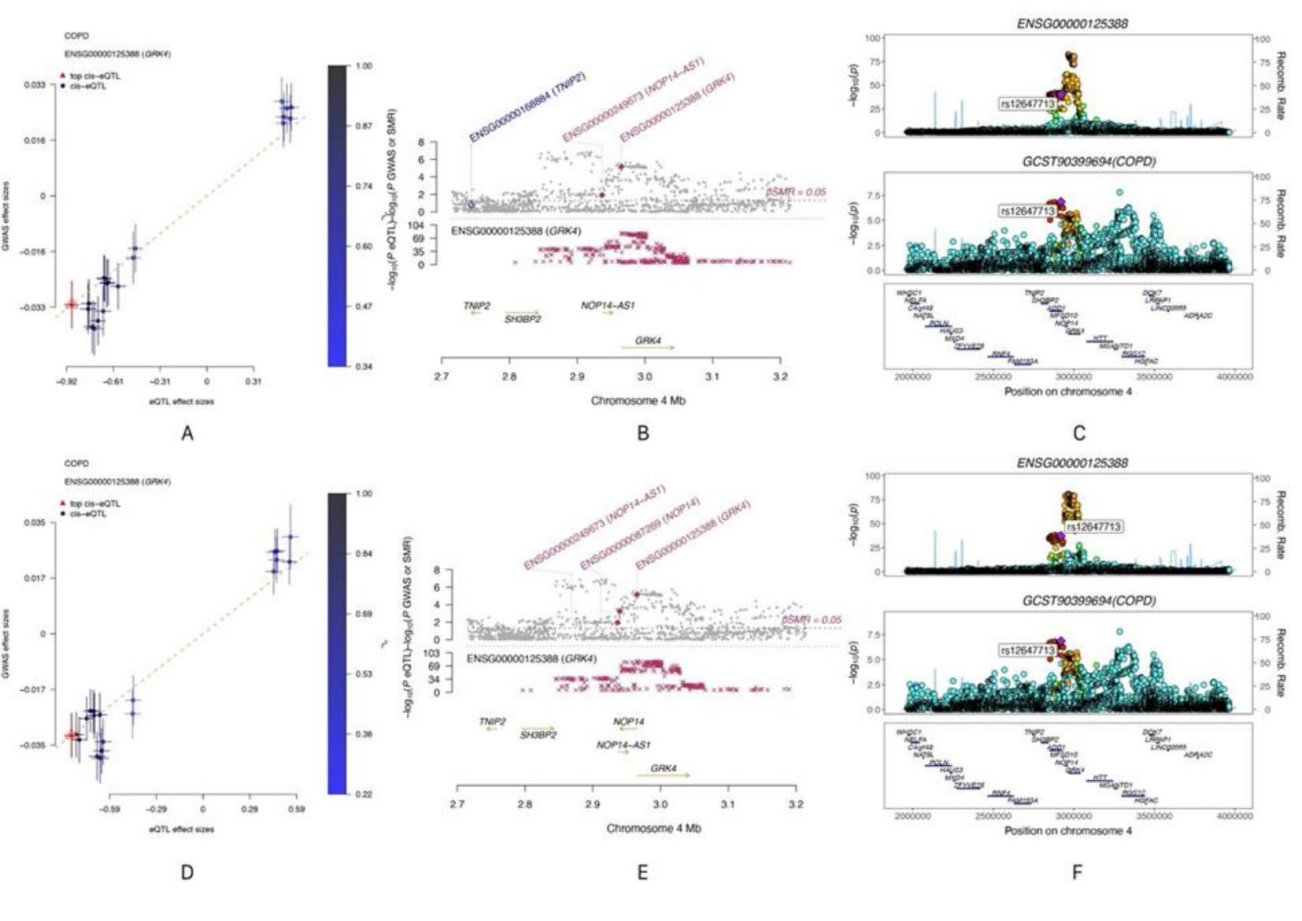
SMR analysis effect and locus plots, and colocalization plots (Note: A, B, C: SMR analysis effect plot, locus plot, and colocalization plot in Artery Tibial; D, E, F: SMR analysis effect plot, locus plot, and colocalization plot in Cells Cultured Fibroblasts).

In subsequent causal validation analyses, we investigated the potential causal roles of these four key candidate genes in COPD using SMR, colocalization, and MR analyses (Supplementary Material 1, S14, S15, and S16). SMR analysis indicated that *GRK4* met the significance threshold in 22 tissues (Fig. 9). Colocalization analysis demonstrated that *GRK4* shared the same genetic signal with COPD, specifically SNP *rs12647713 (*Fig. 10C *and F)*, in the artery tibial and cultured fibroblasts, corroborating the SMR findings (Fig. 10A, B, D, and E).

**Fig. 11:**
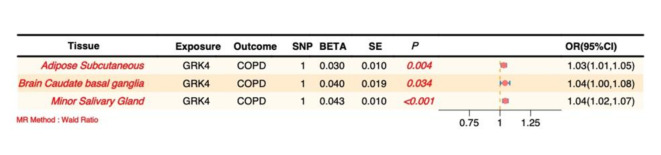
Forest plot of MR analysis.

Moreover, MR analysis showed positive causal effects of *GRK4* on COPD in the adipose subcutaneous tissue, brain caudate basal ganglia, and minor salivary gland (*P* < 0.05, OR > 1) (Fig. 11). These findings were also validated in an independent database, further supporting the hypothesis that *GRK4* is a key susceptibility gene for COPD. Collectively, *GRK4* demonstrated significant associations with COPD across multiple analyses, suggesting its potential involvement in the genetic susceptibility and pathophysiological mechanisms of COPD.

**Table 5:**
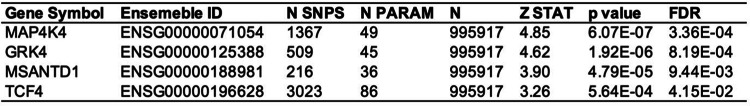
Four candidate genes (*MAP4K4*, *GRK4*, *MSANTD1*, and *TCF4*) in MAGMA.

### GeneMANIA analysis

**Fig. 12:**
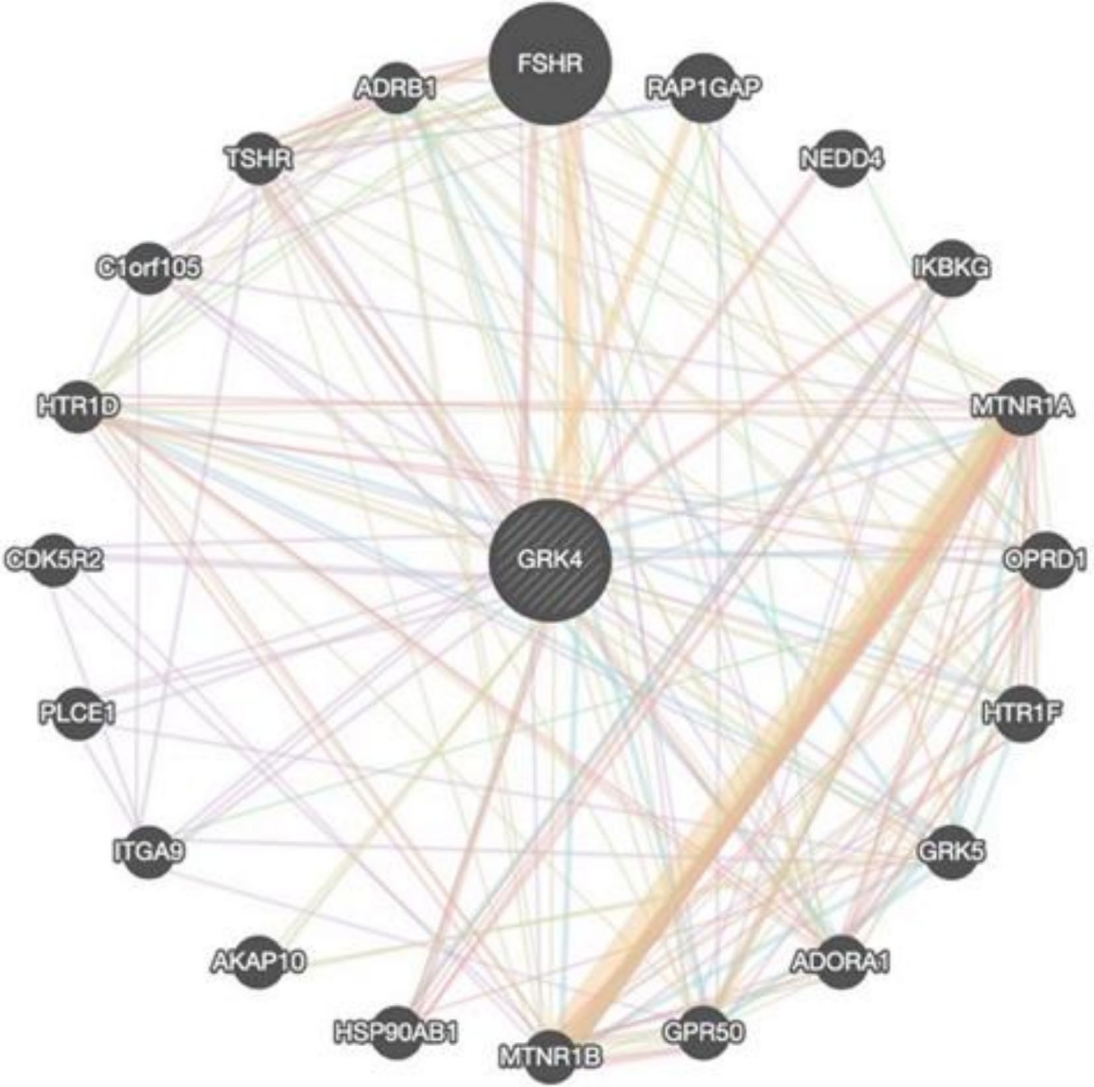
GeneMANIA gene network centered on *GRK4*.

The potential interaction gene network centered around *GRK4* is illustrated in Fig. 12 (Supplementary Materials 3). The most prominent functions within the *GRK4*-associated gene network include the G protein-coupled receptor signaling pathway, coupled to cyclic nucleotide second messengers, G protein-coupled receptor activity, and adenylate cyclase-modulating G protein-coupled receptor signaling pathways (Supplementary Materials 1 S17).

## Discussion

In this study, we systematically evaluated the relationship between genetic susceptibility of gene expression and COPD risk using GWAS data from Finnish R11 and eQTL data from GTEx V8. Through cross-tissue and single-tissue TWAS analyses, as well as MAGMA validation, we identified *GRK4* as a COPD susceptibility gene and further validated its significance using SMR, MR, and colocalization analyses. Moreover, validation in an independent database reinforced the potential role of *GRK4* as a COPD susceptibility gene. Finally, GeneMANIA analysis provided deeper insights into the potential functions of *GRK4*, offering critical directions for future research.

COPD is a complex, multifactorial disease influenced by both environmental factors and genetic susceptibility. For instance, significant variability in COPD incidence among individuals with similar smoking histories suggests a strong association with genetic variations^45^. To date, GWAS and gene expression analyses have identified numerous genes and molecular pathways implicated in COPD pathogenesis. Notably, SNPs at the α-nicotinic acetylcholine receptor (*CHRNA3/5*) locus have been shown to be significantly associated with COPD risk and lung function, with these findings validated in multiple independent cohorts^46^. Furthermore, research by Michael H. Cho et al.^47^ identified a novel COPD susceptibility locus, *19q13*, through a GWAS involving 3,499 COPD cases and 1,922 controls across four distinct cohorts, with additional validation in familial datasets. These studies provide valuable genetic targets for early screening and personalized treatment strategies for COPD.

With the gradual elucidation of the molecular mechanisms underlying COPD, the G protein-coupled receptor kinase (GRK) family has emerged as a potential contributor to its pathogenesis. GRKs, as serine/threonine protein kinases, have gained significant attention due to their pivotal roles in signal transduction. The GRK family is divided into two main subfamilies: *GRK2/3* and *GRK4/5/6*^48^. GRKs interact with agonist-activated G protein-coupled receptors (GPCRs), influencing receptor phosphorylation and thereby modulating signal transmission and desensitization^49^. During the pathological process of inflammatory responses, the expression levels of GRKs change notably. Studies have shown that GRK membrane activity, as well as the expression of *GRK2* and *GRK5*, is markedly increased in IL-1-treated rat lung tissue, with these changes being completely reversed following treatment with the anti-inflammatory steroid dexamethasone^50^. Furthermore, lipopolysaccharide (LPS) signaling via the TLR4 pathway has been found to downregulate *GRK2* and *GRK5* expression in polymorphonuclear neutrophils (PMNs)^51^. Similarly, significant downregulation of *GRK2* and *GRK6* expression has been observed in peripheral blood mononuclear cells (PBMCs) from patients with rheumatoid arthritis (RA) or multiple sclerosis (MS)^52–54^.

Currently, studies directly investigating *GRK4* in COPD are limited; however, there is substantial evidence linking *GRK4* genetic variation to hypertension, where it plays a critical role in regulating receptor expression and function related to blood pressure. By modulating sodium handling in the kidneys, arterial function, and blood pressure control, *GRK4* inhibition can restore normal blood pressure regulation^55^. This evidence suggests that *GRK4* may similarly influence COPD pathophysiology through analogous signaling mechanisms. It is well-established^56^ that cardiovascular disease significantly contributes to the onset and progression of COPD, with both conditions sharing risk factors such as smoking, low socioeconomic status, and sedentary lifestyle. Notably, COPD patients have a higher prevalence of diabetes and hypertension compared to healthy individuals, a trend particularly pronounced in the GOLD (Global Initiative for Chronic Obstructive Lung Disease) stage III and IV subgroups of the Atherosclerosis Risk in Communities (ARIC) study^57^. Given the interplay between COPD and hypertension, it is plausible that *GRK4* may play a cross-functional role in the pathogenesis of both conditions via shared pathophysiological pathways. One of the hallmarks of COPD is elevated oxidative stress^58^, which is crucial in lung tissue damage and sustained inflammation. Recent studies have shown that *GRK4* expression is inherently upregulated in the kidneys and arteries of patients with essential hypertension and is significantly influenced by environmental factors such as cold stress, particulate matter (PM) exposure, and infection. These factors induce an increase in reactive oxygen species (ROS) levels, thereby upregulating *GRK4* expression and affecting oxidative stress-related signaling pathways. Long-term exposure to PM2.5 has been demonstrated to increase ROS production, blood pressure, and *GRK4* expression in Sprague-Dawley (SD) rats. This phenomenon is closely associated with oxidative stress, and the administration of the antioxidant Tempol, which inhibits ROS production, significantly reduces *GRK4* expression in the kidneys, alleviating excessive phosphorylation of the D1 receptor (D1R). This process not only improves sodium excretion but also effectively lowers blood pressure in PM2.5-exposed SD rats, further underscoring the crucial role of oxidative stress in *GRK4* regulation^59,60^. This mechanism may also be relevant to COPD pathogenesis, where *GRK4* could influence the disease through the regulation of oxidative stress and chronic inflammatory responses. Therefore, *GRK4* likely exerts significant roles beyond the cardiovascular system, potentially impacting COPD through shared physiological pathways. Future research should focus on the cross-system regulatory role of *GRK4* in the cardiopulmonary system, particularly its specific functions in oxidative stress and inflammatory responses. Unraveling *GRK4*’s role in COPD could provide valuable insights for developing novel therapeutic targets.

Given the known role of *GRK5* in regulating inflammatory responses and the general involvement of the GRK family in inflammation, we hypothesize that *GRK4* may modulate the inflammatory response in COPD by influencing oxidative stress, thereby contributing to disease onset and progression. However, significant limitations persist in current research. Firstly, the specificity of *GRK4*’s function remains unclear. Although it may be involved in the inflammatory response, the precise regulatory mechanisms are yet to be elucidated and may be influenced by multiple factors. Secondly, many studies rely on model systems; results from animal and cell experiments may not fully represent the true pathological state in humans. Lastly, there may be redundancy and functional complementarity among GRK family members. Even if *GRK4* plays a pivotal role in COPD, designing specific therapeutic strategies targeting it poses challenges.

In light of these limitations, we propose several directions for future research. First, validating the specific role of *GRK4* in COPD through gene knockout or overexpression studies in cell and animal models, focusing on its impact on the expression of inflammatory markers, immune cell migration, and lung function changes. Second, employing molecular docking and molecular dynamics simulations^61,62^ to analyze interactions between *GRK4* and associated pathways, exploring how these pathways might influence inflammatory responses in COPD. Finally, assessing *GRK4* expression levels in samples from COPD patients to investigate its correlation with disease severity or prognosis, thereby validating its potential as a therapeutic target. These efforts would provide a foundation for evaluating the feasibility of *GRK4* as a target for COPD treatment and offer support for future targeted therapeutic strategies.

## Conclusions

This study systematically evaluated the potential role of *GRK4* in COPD susceptibility, incorporating cross-tissue analysis and multi-database validation to reveal that *GRK4* may play a critical role in the pathophysiological processes of COPD, particularly in oxidative stress and inflammation regulation pathways. This finding not only broadens our understanding of the systemic mechanisms underlying COPD but also offers new perspectives and entry points for multi-level, cross-tissue research on the disease. The results of this study provide essential evidence for future genetic marker screening in COPD, suggesting that *GRK4* could be a valuable candidate for early detection, risk assessment, and targeted intervention. Moreover, the functional characteristics of *GRK4* and its potential synergistic interactions with other GRK family members present new avenues for exploring the complex pathogenesis of COPD. Future research should focus on clarifying the specific regulatory pathways of *GRK4* in oxidative stress and inflammatory responses through gene editing and molecular mechanism analysis and examining its cross-system impact in COPD and coexisting cardiovascular diseases. Overall, this study supports *GRK4* as a potential target for personalized treatment strategies for COPD and lays the groundwork for the development of novel targeted therapies. Future investigations should aim to validate *GRK4* expression changes at different stages of COPD and assess its clinical relevance, thereby solidifying its application as a therapeutic target and providing a scientific basis for precise treatment and intervention strategies.

## Electronic supplementary material

Below is the link to the electronic supplementary material.

Supplementary Material 1

Supplementary Material 2

Supplementary Material 3

## Data Availability

Data are publicly available.
