# Supplementary material for: A cross-tissue transcriptome-wide association study reveals GRK4 as a novel susceptibility gene for COPD
Source: Sci Rep. 2024 Nov 18;14:28438. doi: 10.1038/s41598-024-80122-w (PMC11574126; doi:10.1038/s41598-024-80122-w)
Supplement: Supplementary file 3 — Supplementary Material 3 [file 41598_2024_80122_MOESM3_ESM.docx]

COJO Analysis Plot:

Upper Panel:This panel illustrates the distribution and positions of genes along the corresponding chromosomal segment (represented by the horizontal axis). Genes marked in blue and green indicate significant genes identified in the FUSION TWAS analysis (PFDR<0.05). Genes marked in gray are those found to be non-significant or not included in the TWAS results list. In the TWAS analysis, genes marked in blue are relatively less significant, classified as marginally significant genes (marginally TWAS associated genes). Genes marked in green represent the most significant genes within the specific chromosomal region (jointly significant genes).

Lower Panel:The Manhattan plot demonstrates the reduction in significance of the loci associated with blue-marked genes in the GWAS data. The horizontal axis represents chromosomal segments, while the vertical axis shows the p-values of each SNP in the GWAS data, transformed to the -log10 scale. Each point corresponds to a single SNP:

Gray points indicate loci that are significant without accounting for the predicted expression level of the green gene.

Blue points indicate that, after adjusting for the predicted expression level of the green gene, the significance of these loci is reduced from significant to non-significant. This reduction in significance, upon considering the green gene, suggests that the green gene's expression level explains part of the genetic variation, thereby diminishing the statistical significance of these loci.

TWAS Locus Association Plot on Chromosome 4:

| 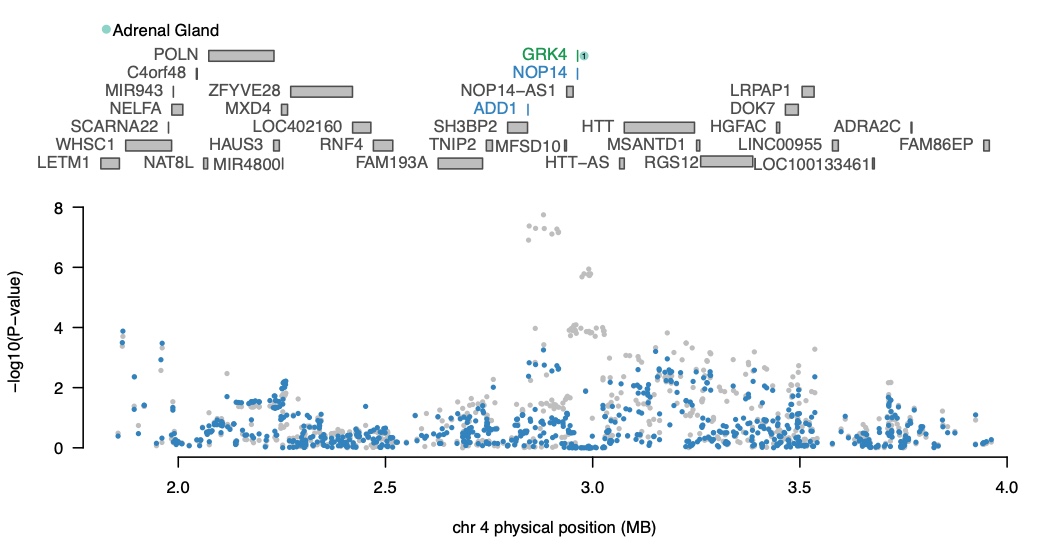 |
| --- |
| 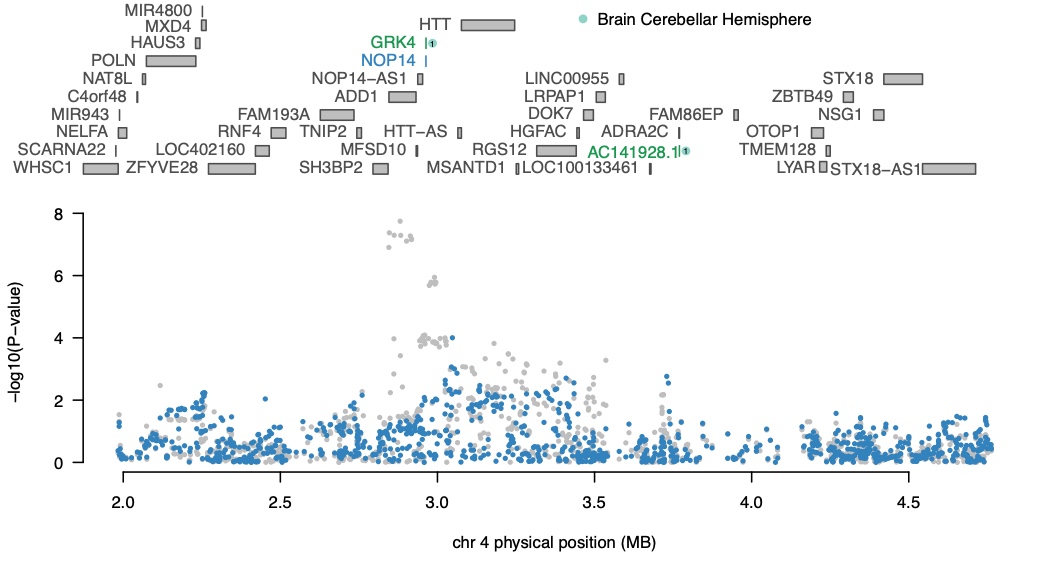 |
|  |
| 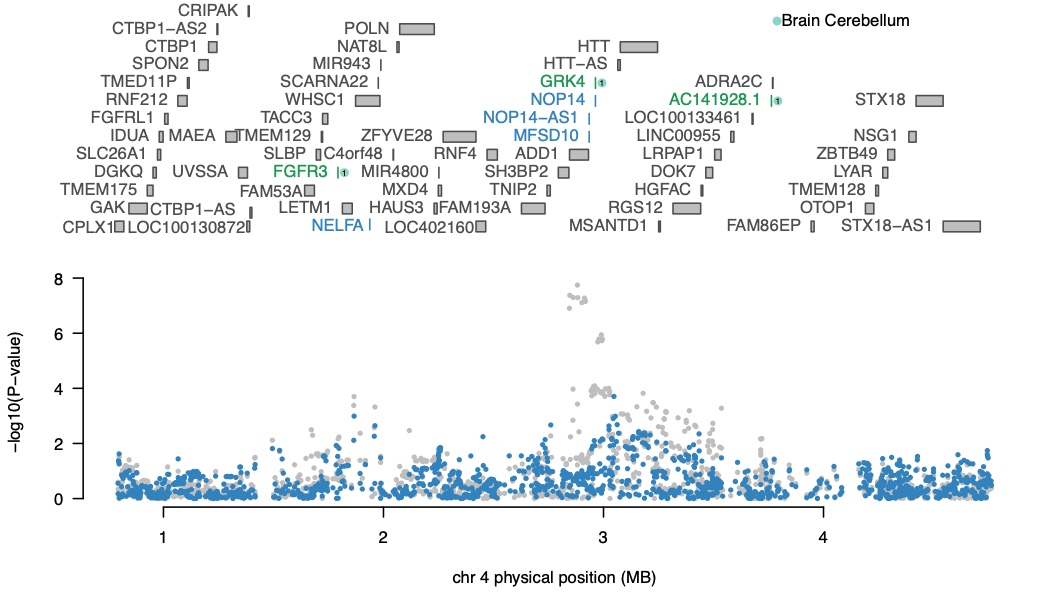 |
|  |
| 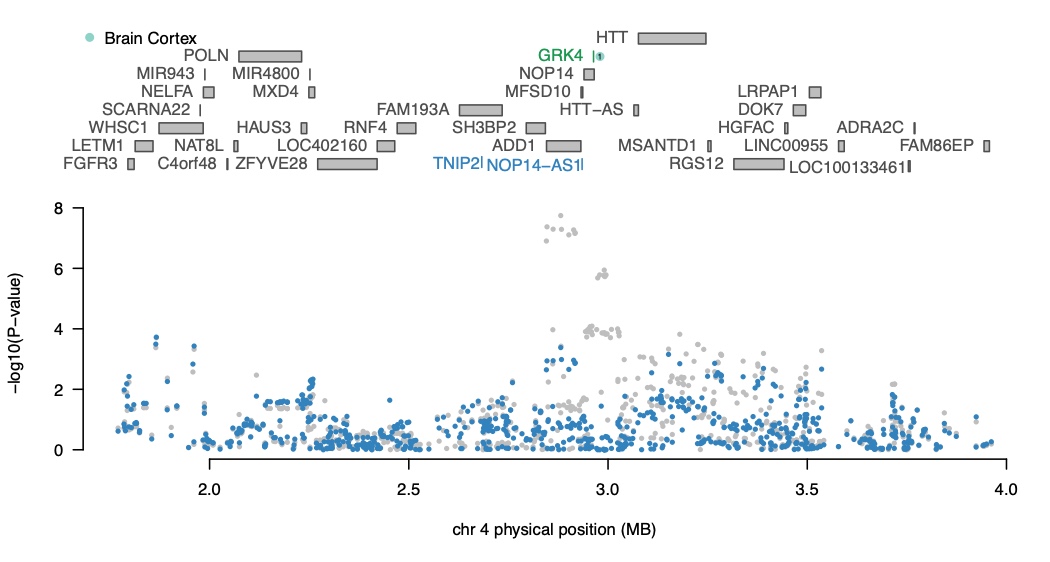 |
|  |
| 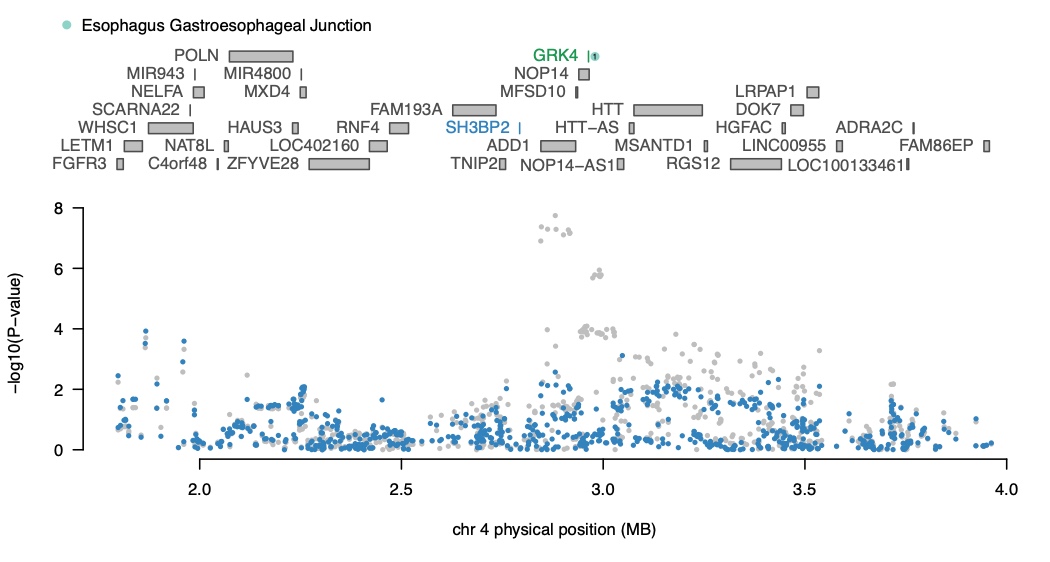 |
|  |
| 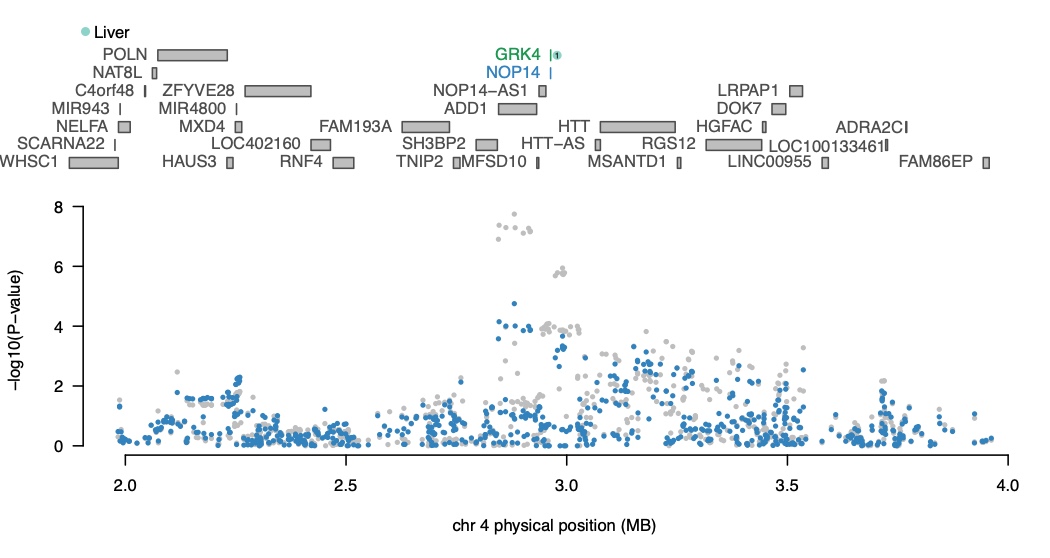 |
|  |
| 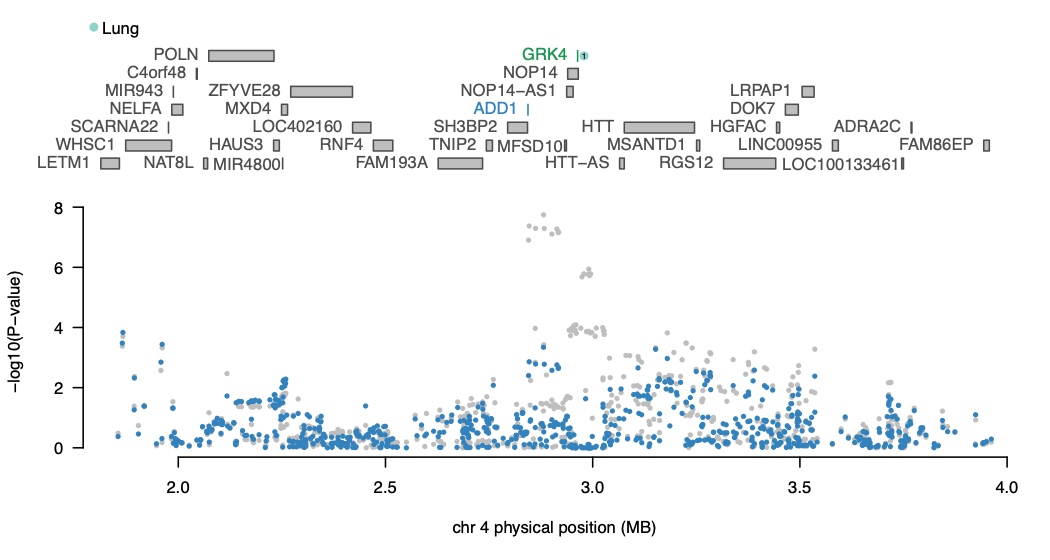 |
|  |
| 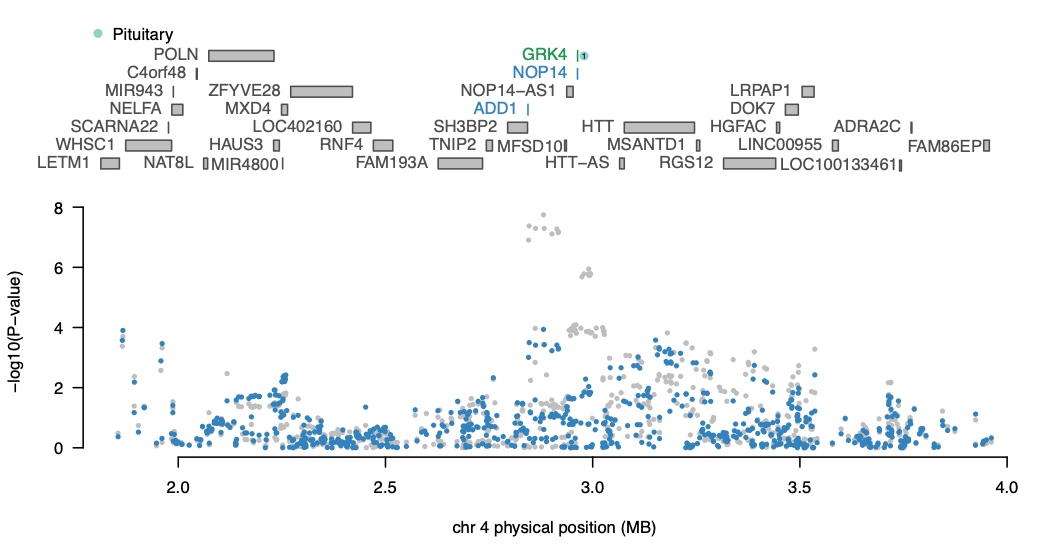 |
|  |
| 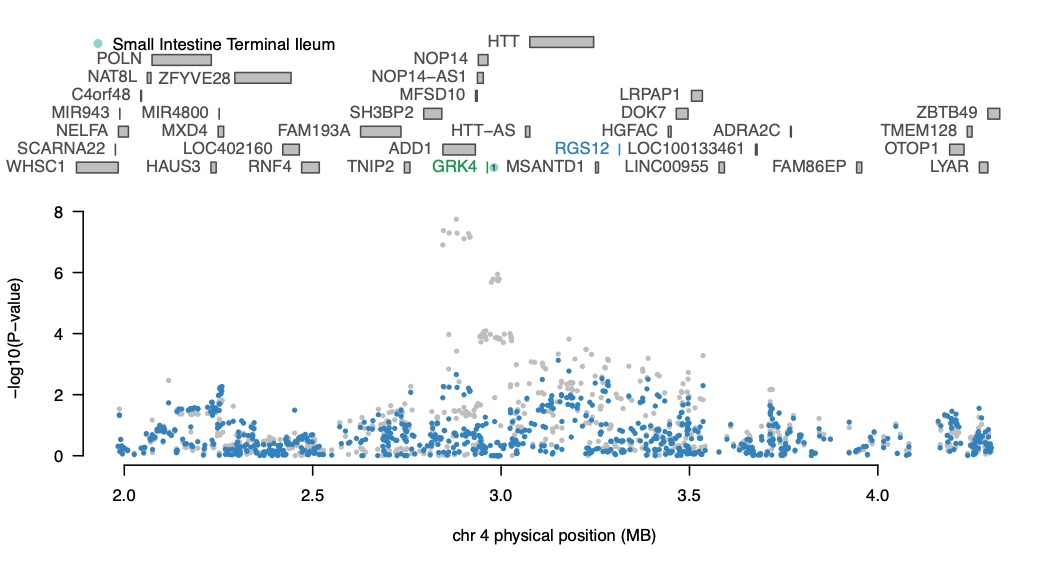 |
|  |
| 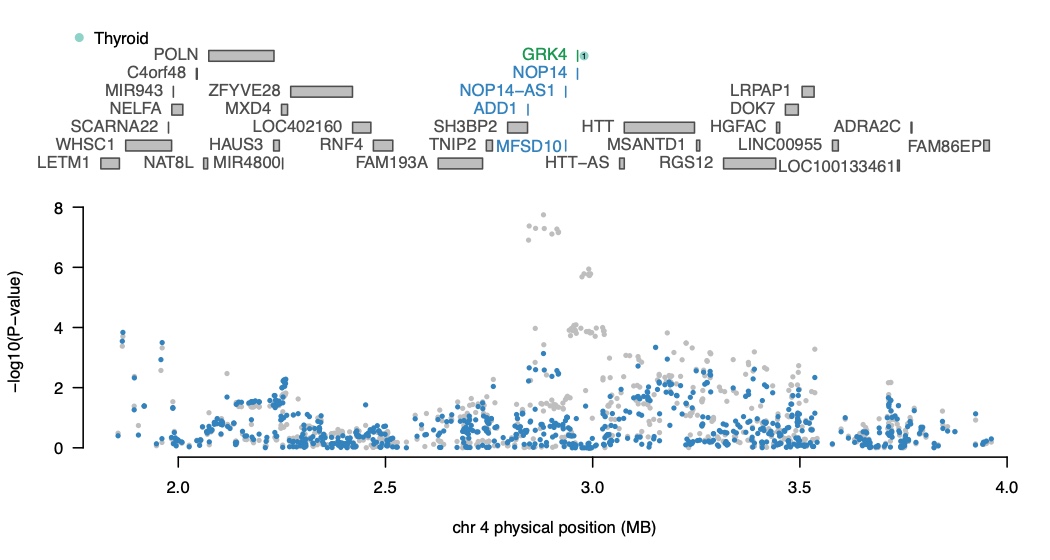 |
|  |

TWAS Locus Association Plot on Chromosome 22:

| 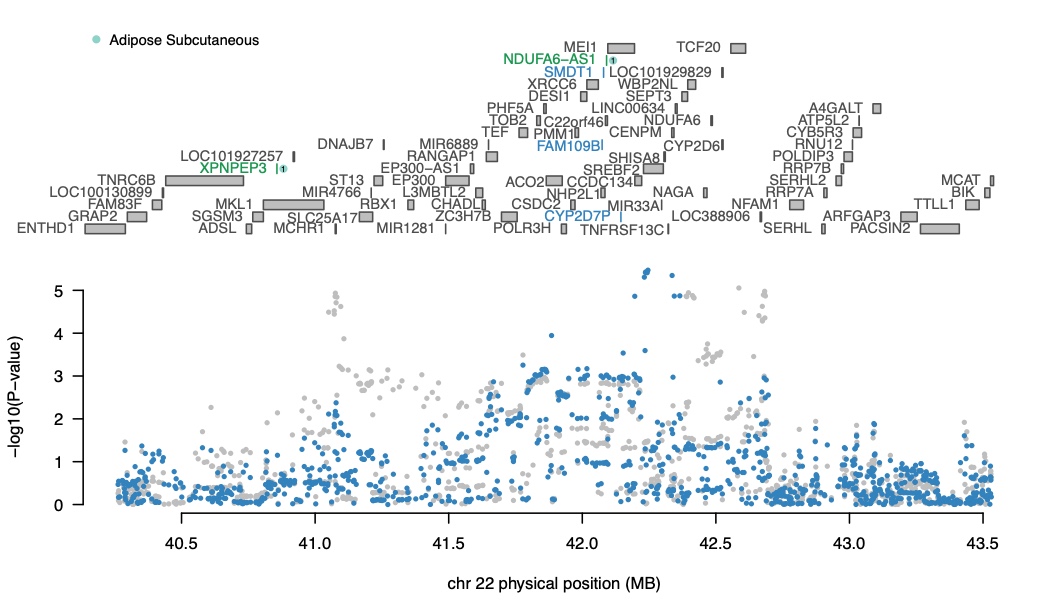 |
| --- |
|  |
| 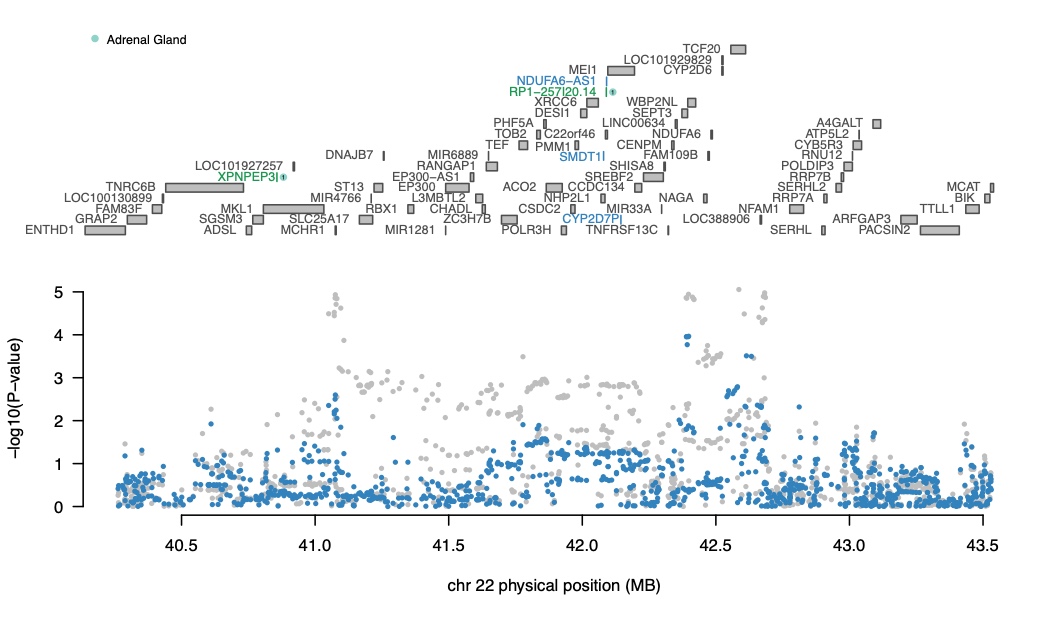 |
|  |
| 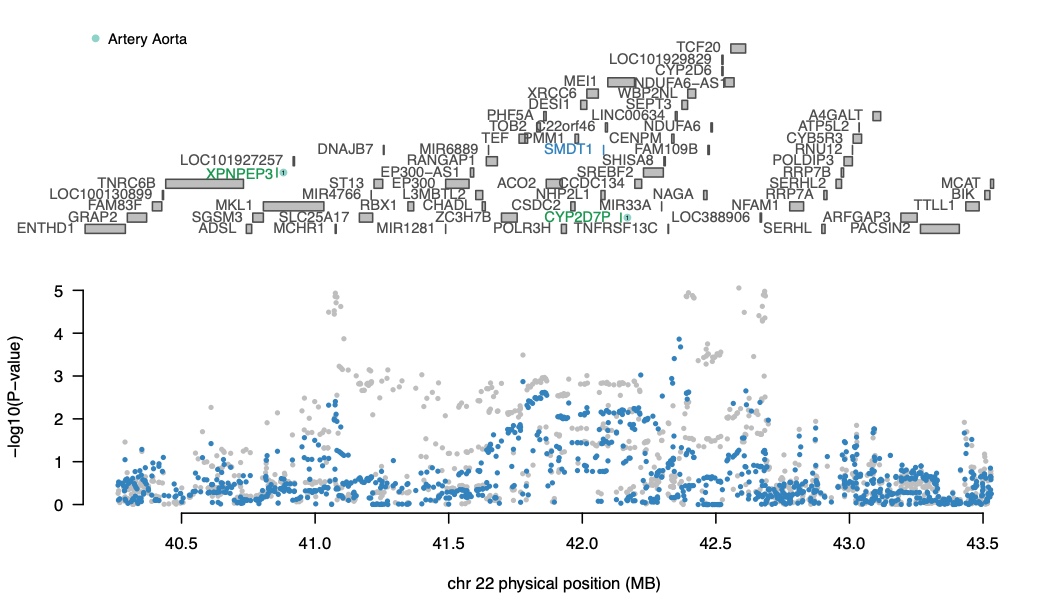 |
|  |
| 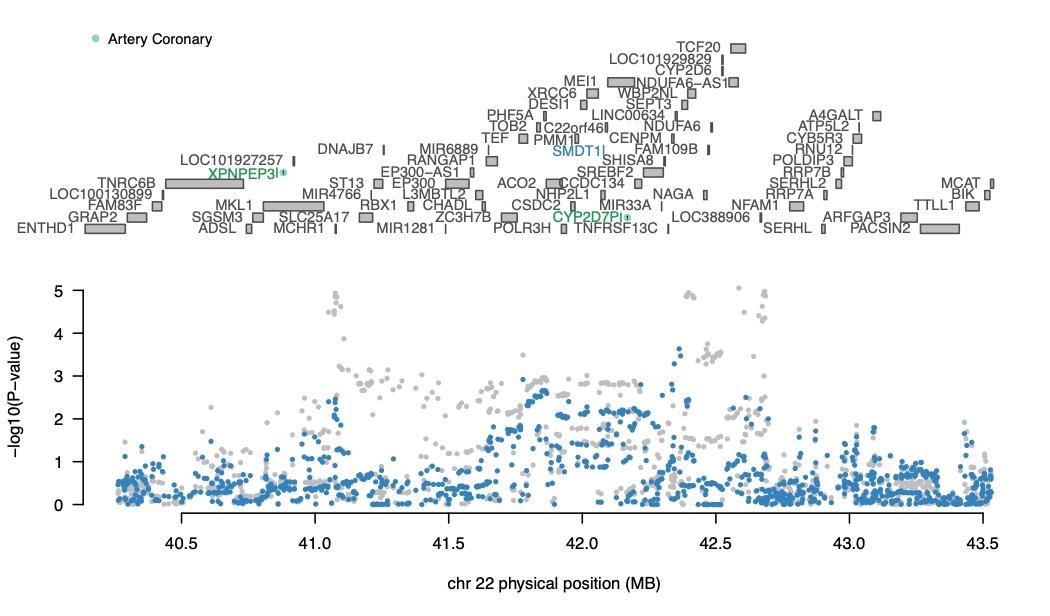 |
| 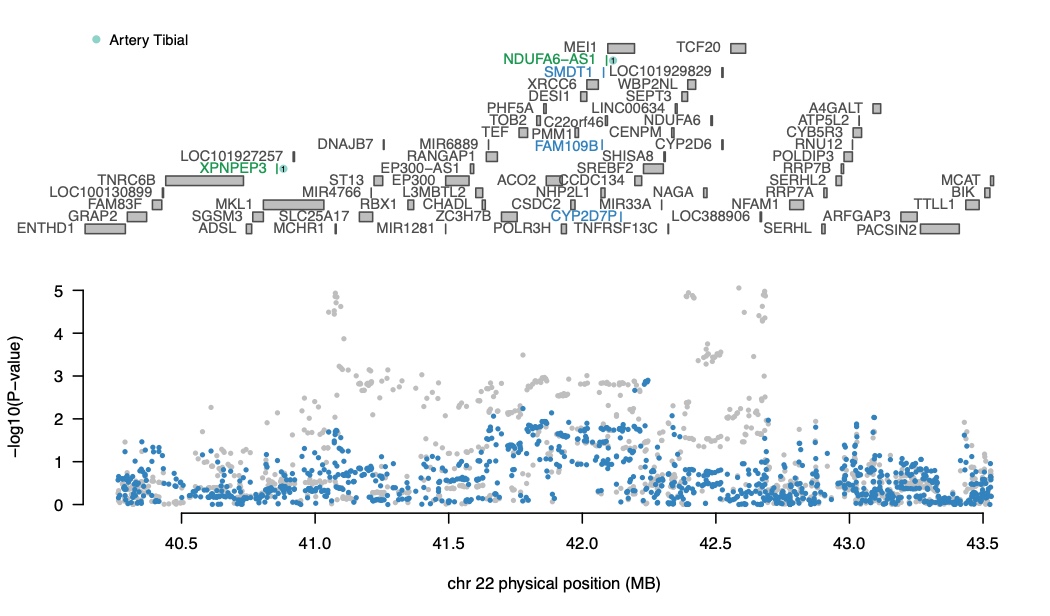 |
| 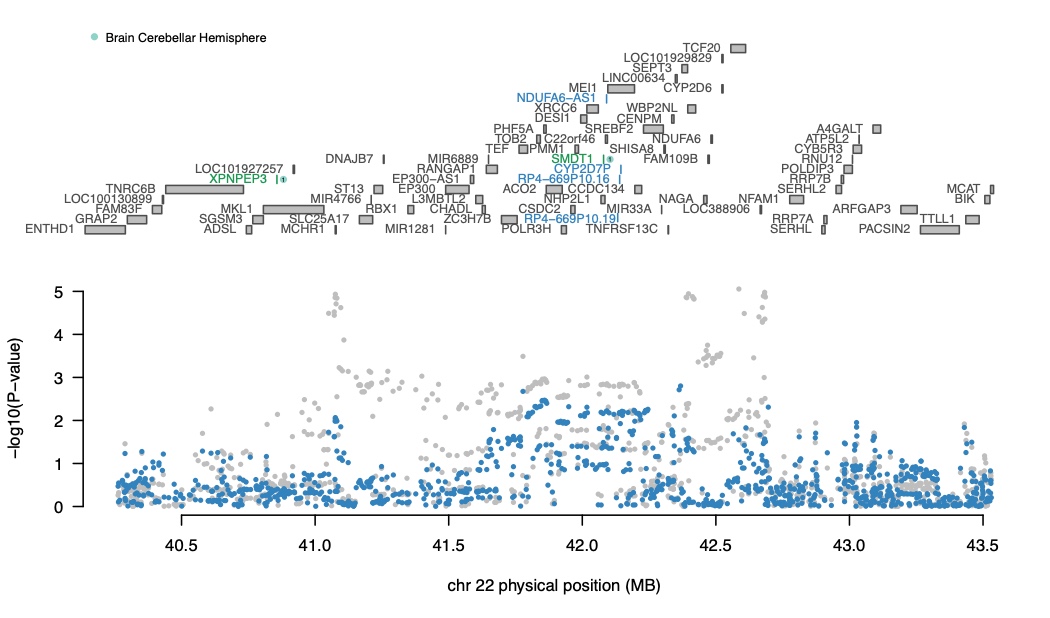 |
|  |
| 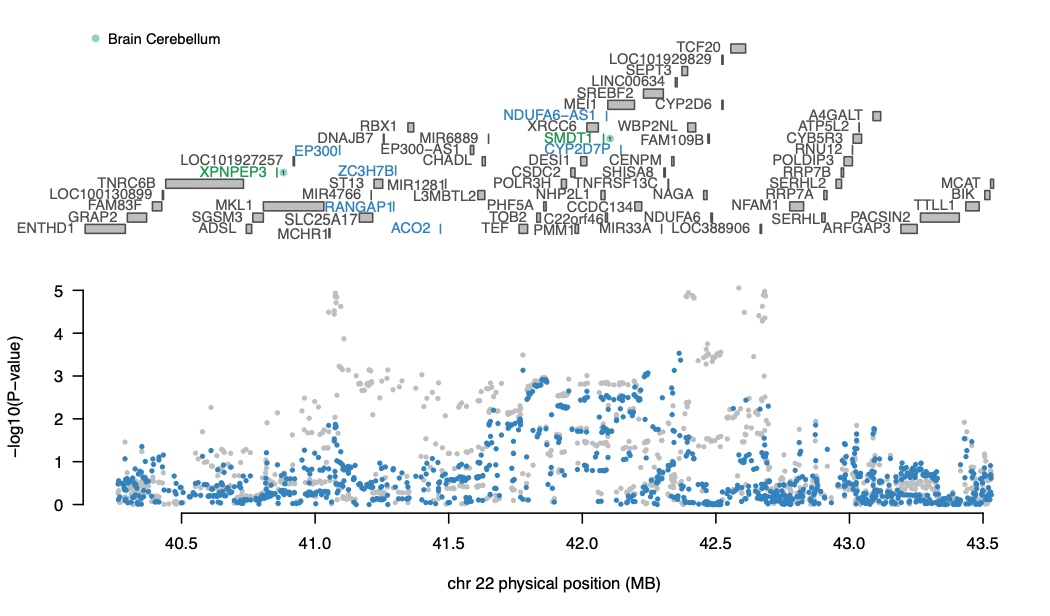 |
|  |
| 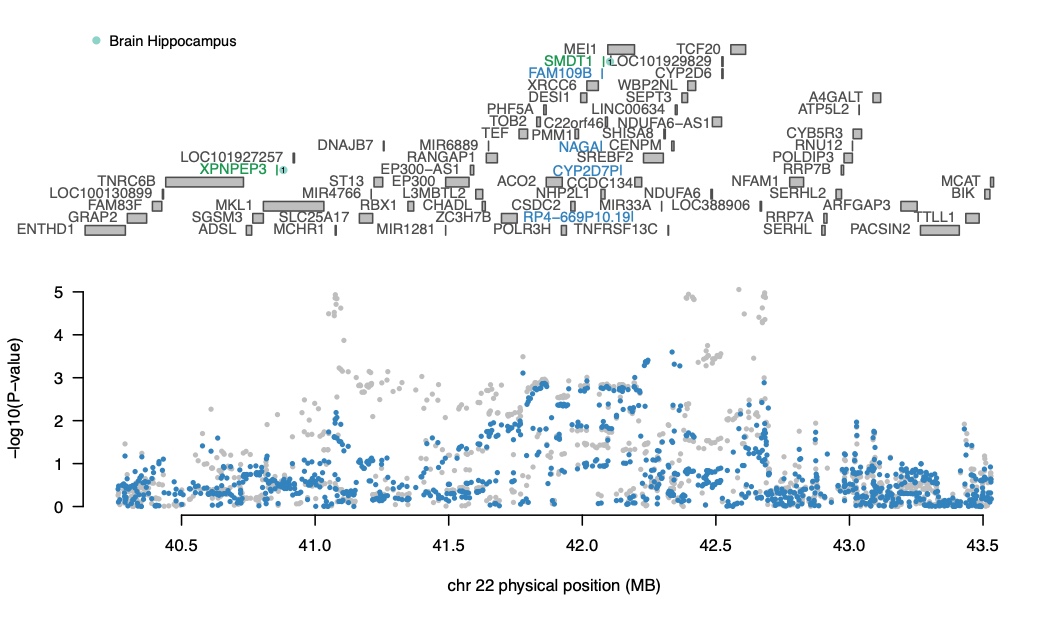 |
|  |
| 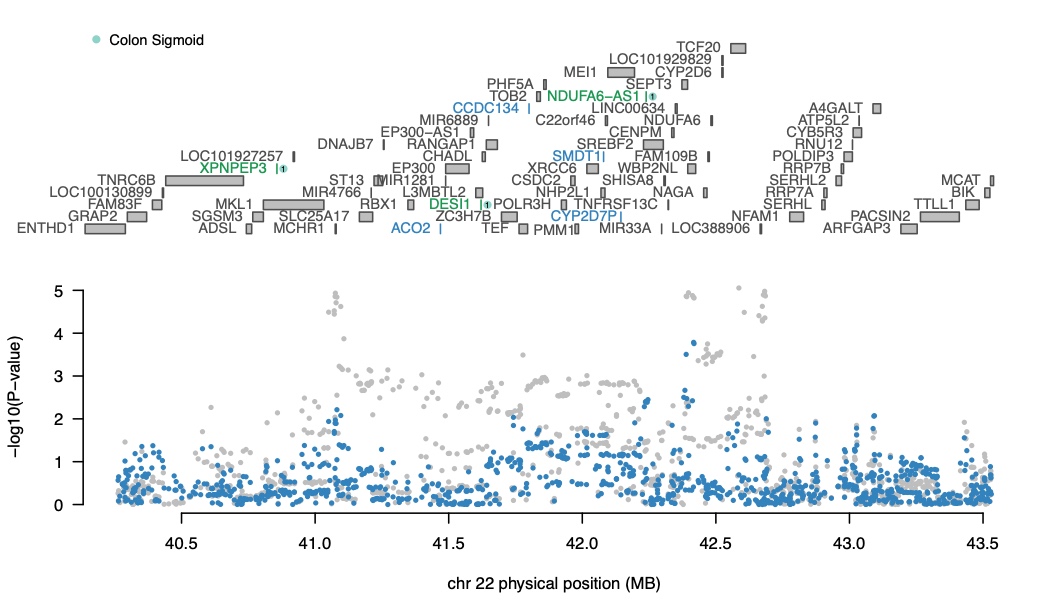 |
|  |
| 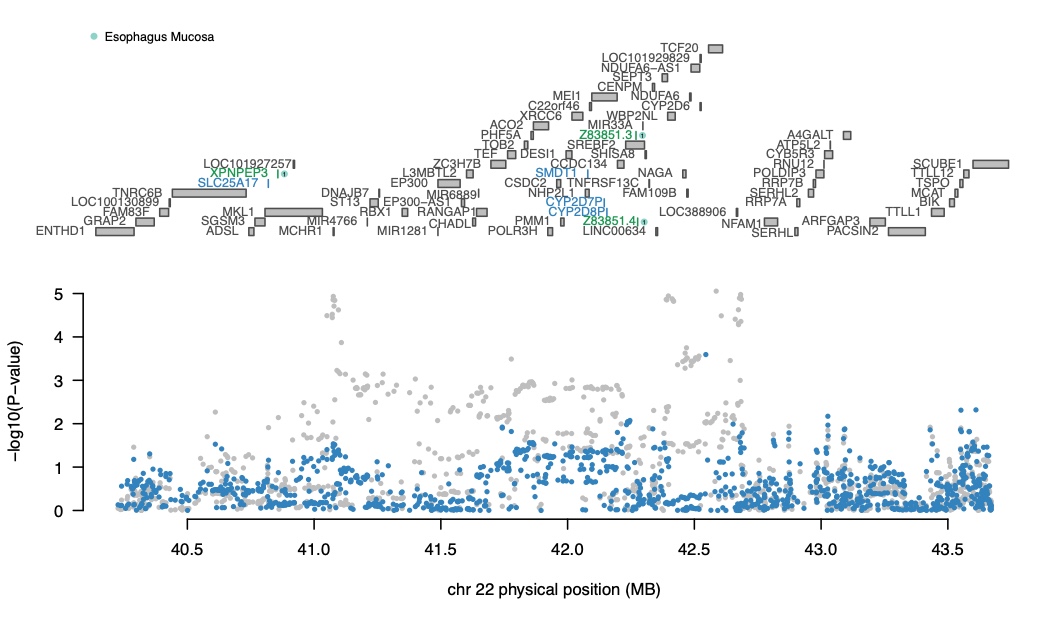 |
|  |
| 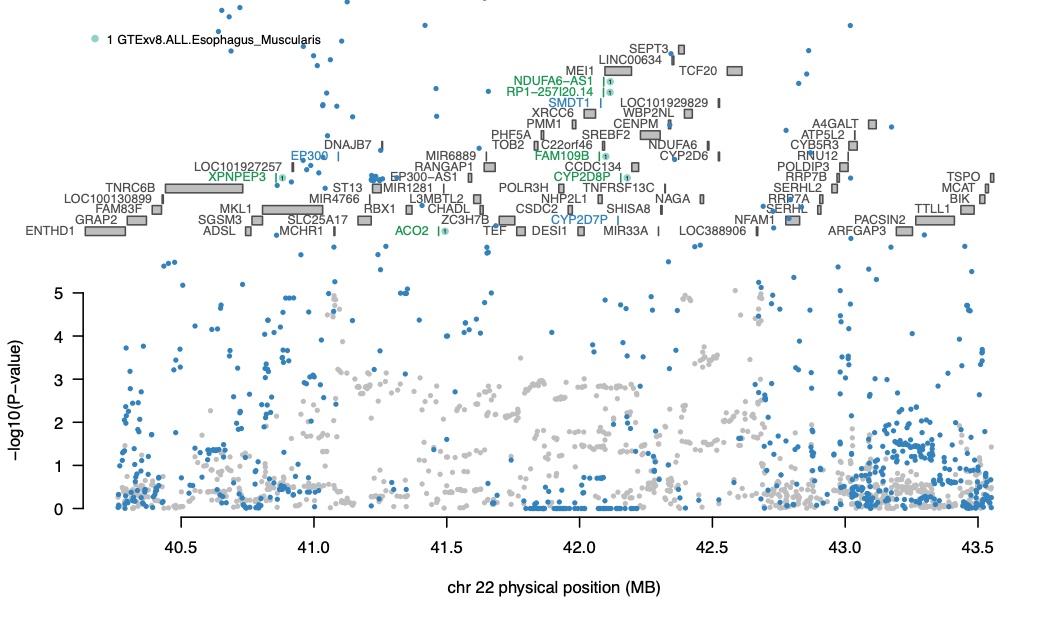 |
|  |
| 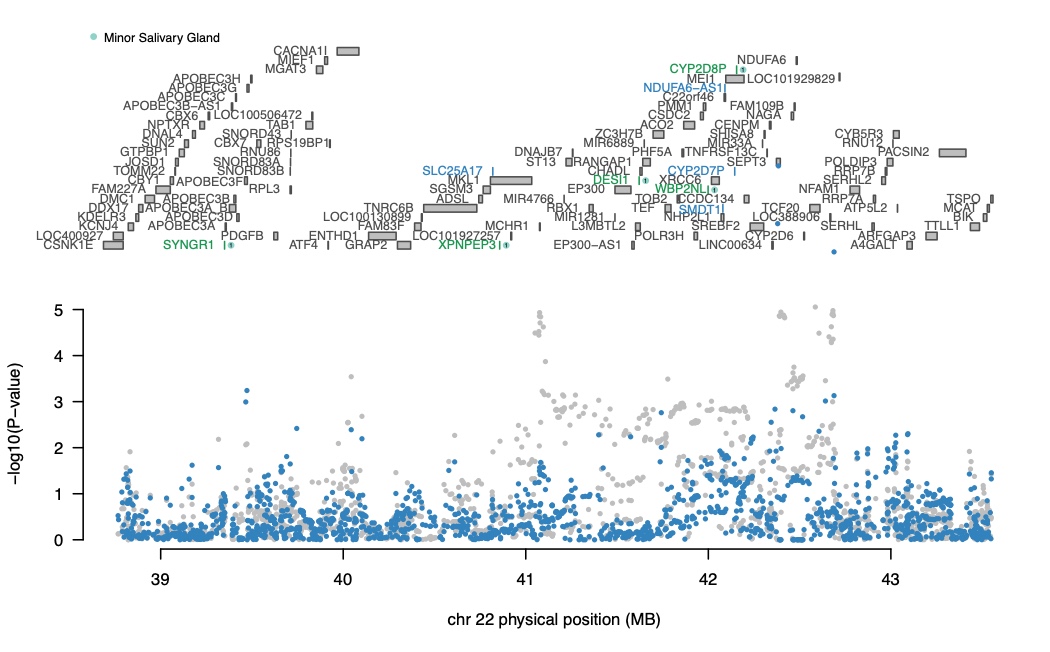 |
|  |
| 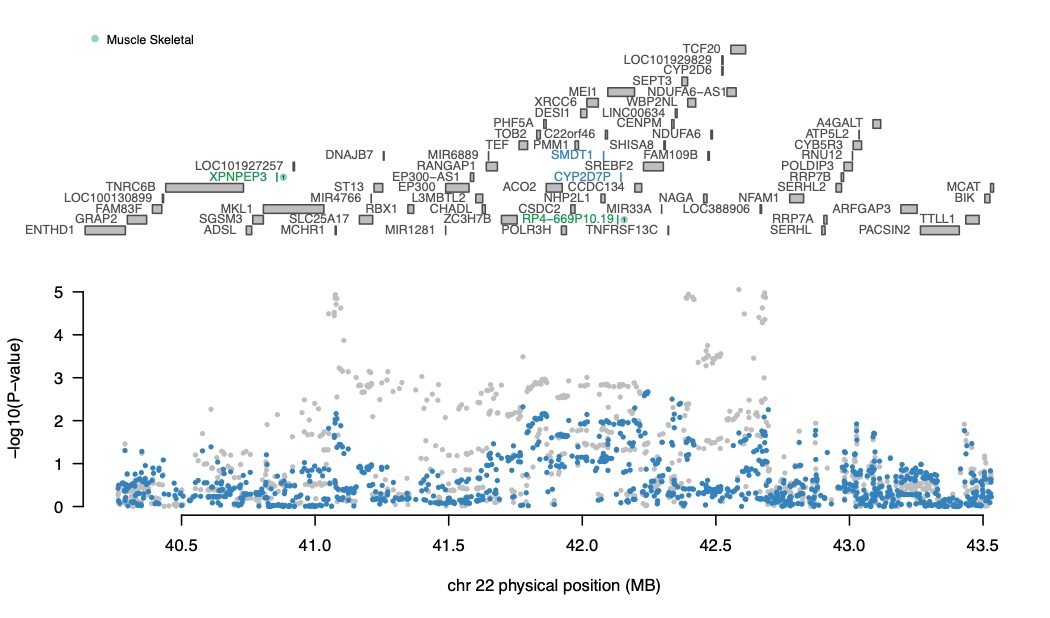 |
|  |
| 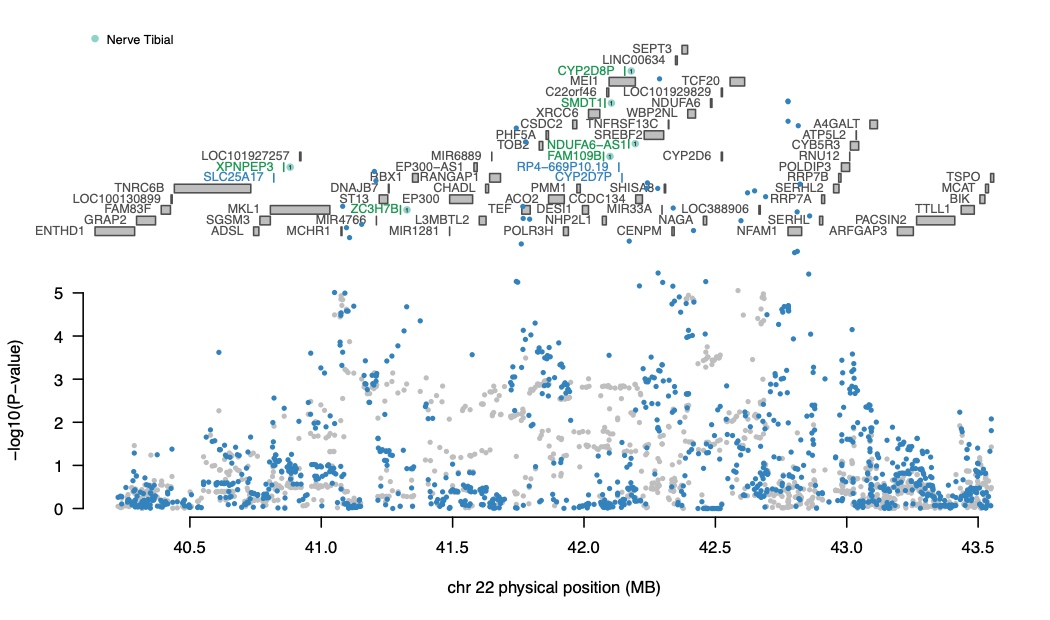 |
|  |
| 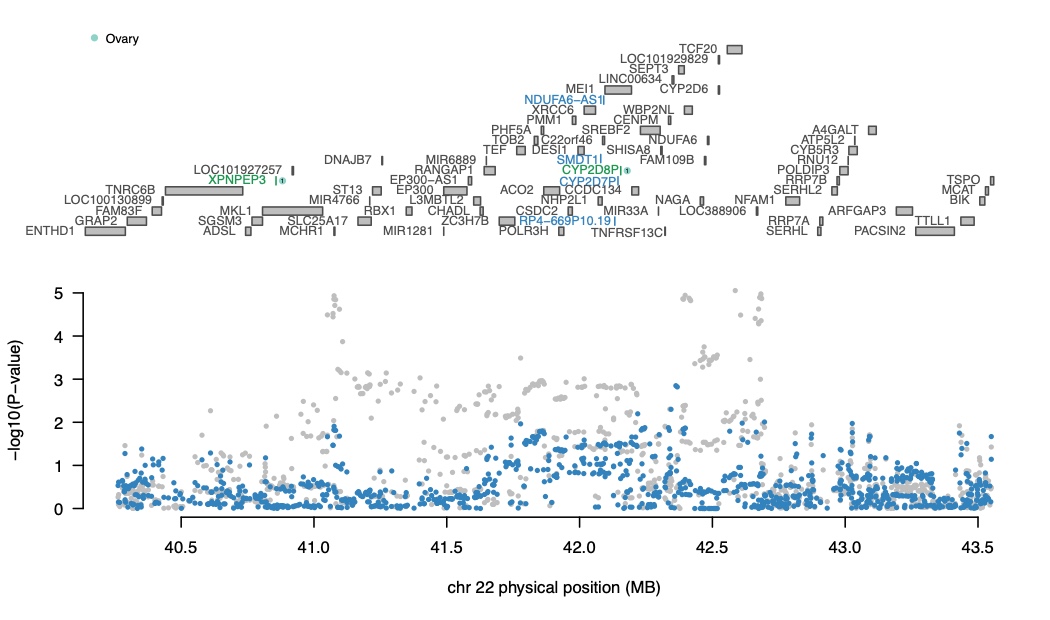 |
|  |
| 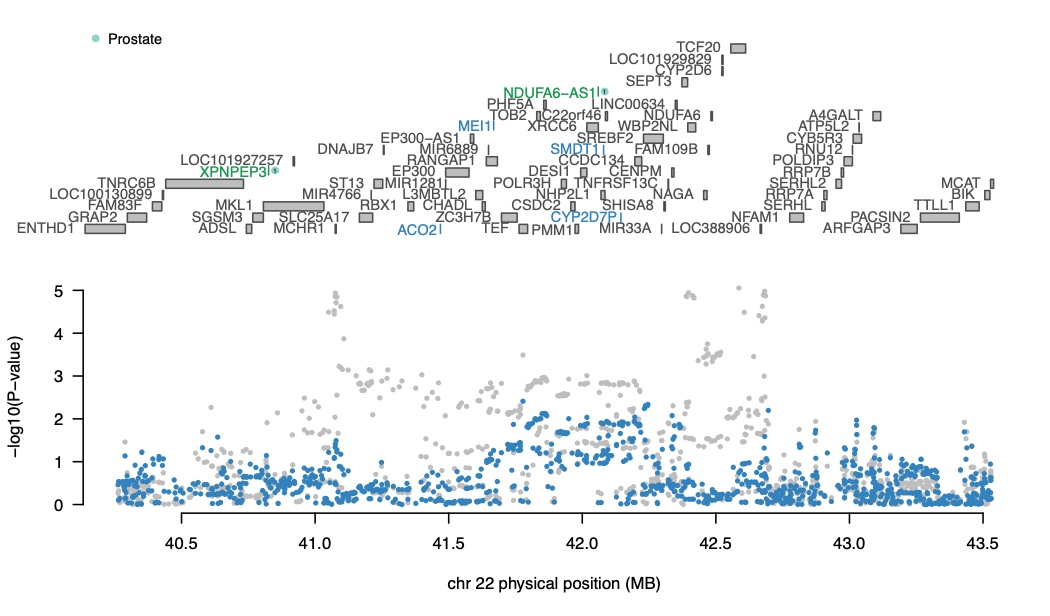 |
|  |
| 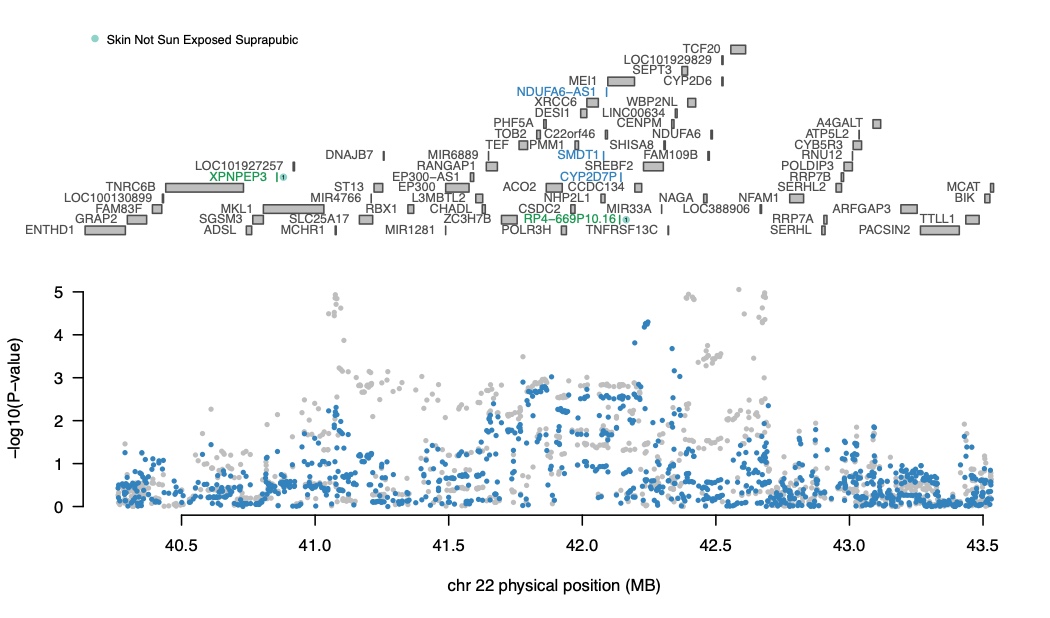 |
|  |
| 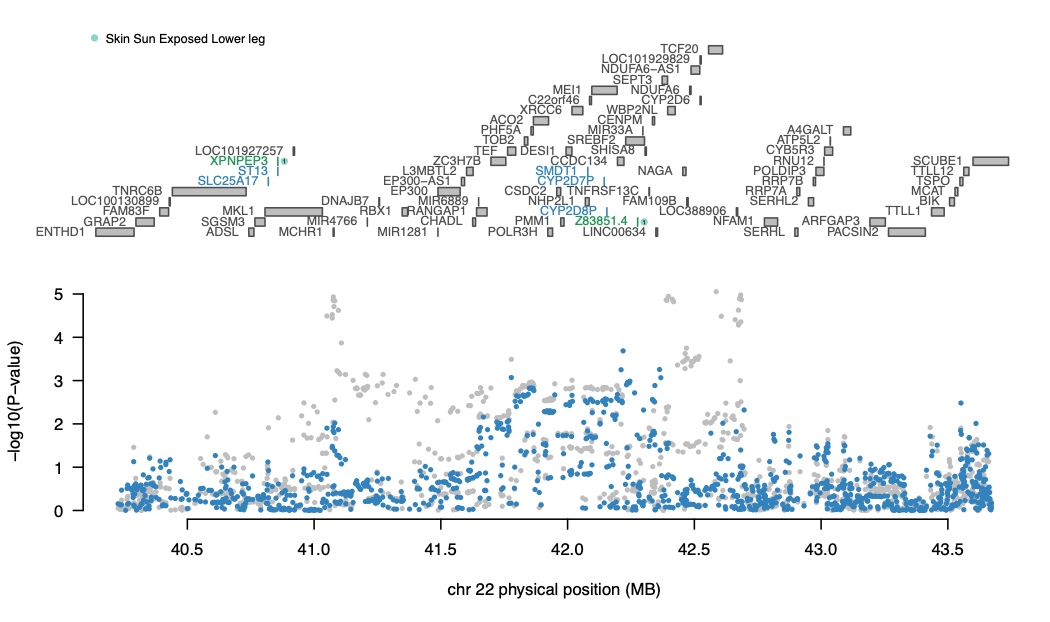 |
|  |
| 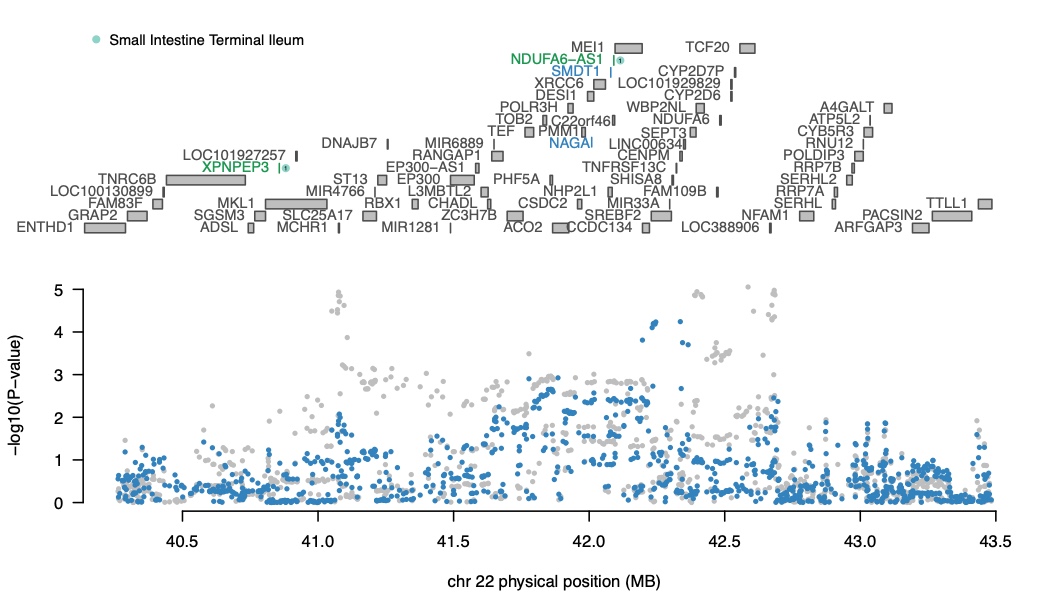 |
|  |
| 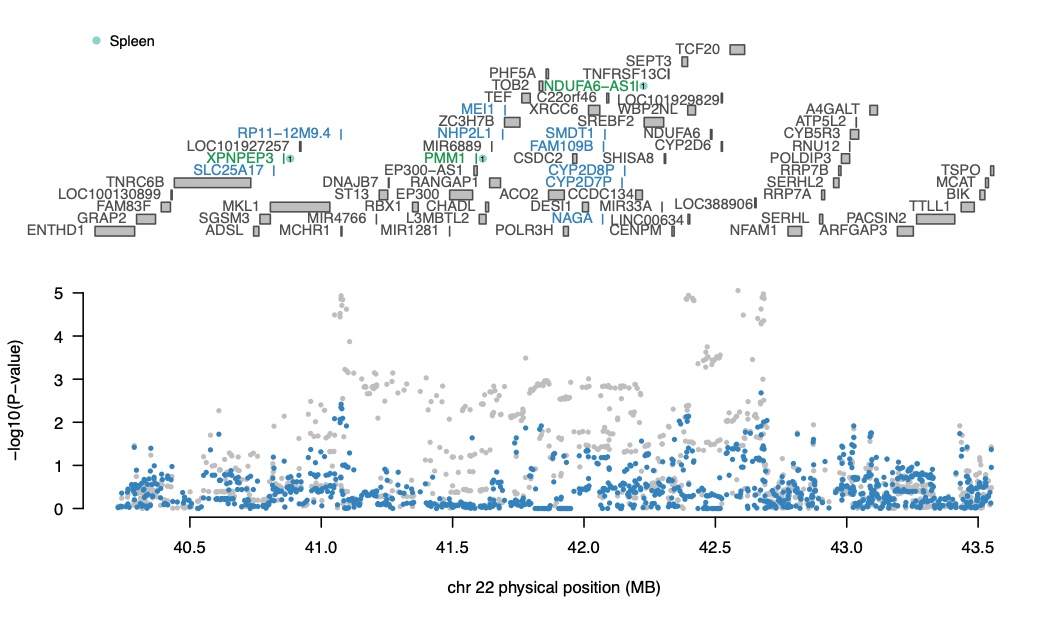 |
|  |
| 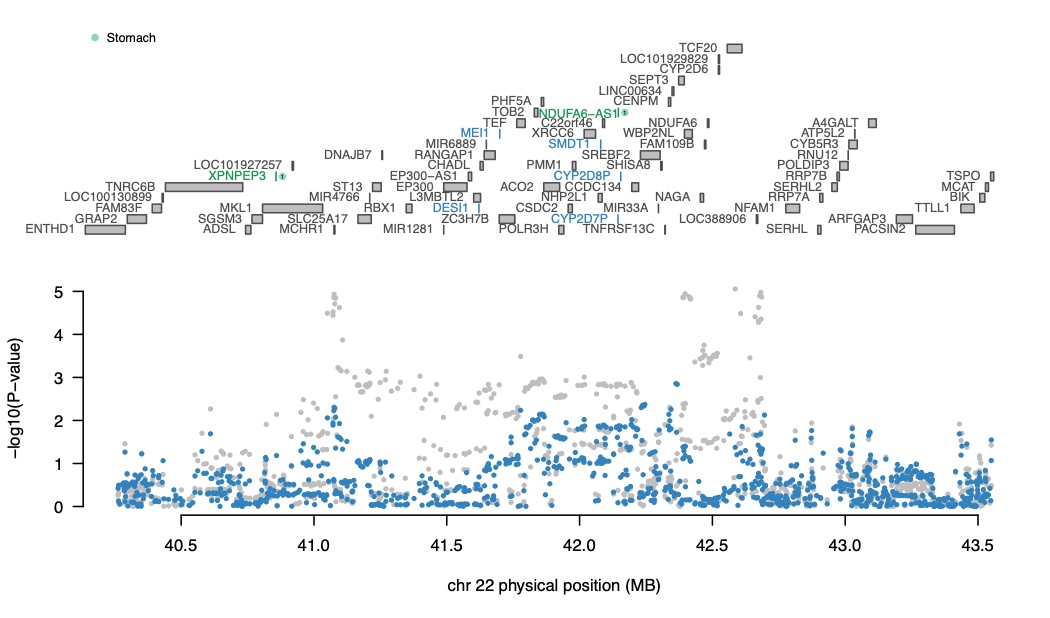 |
|  |
| 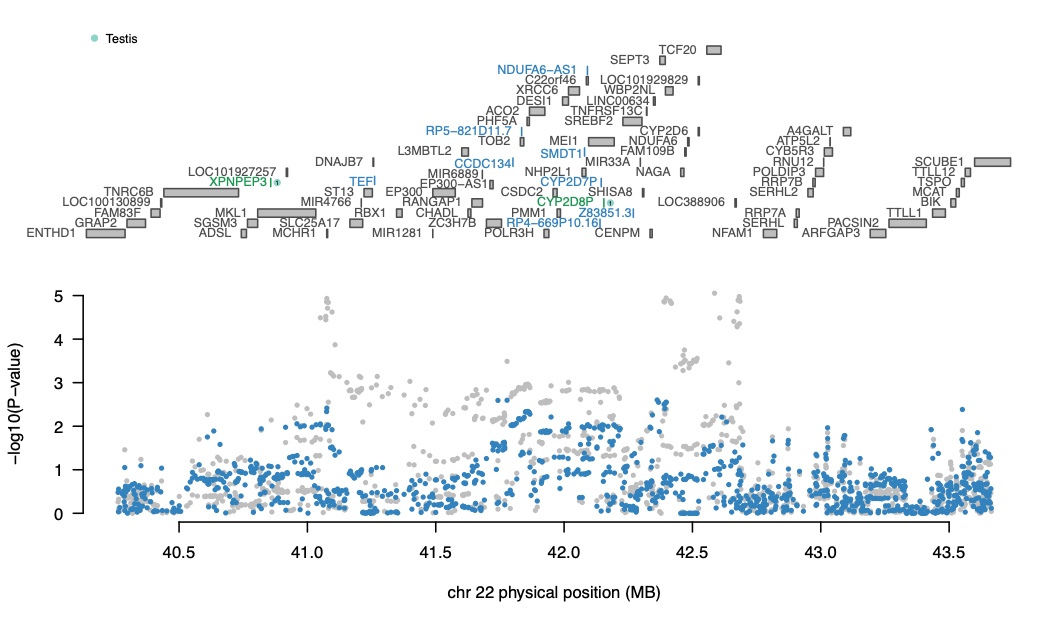 |
|  |
| 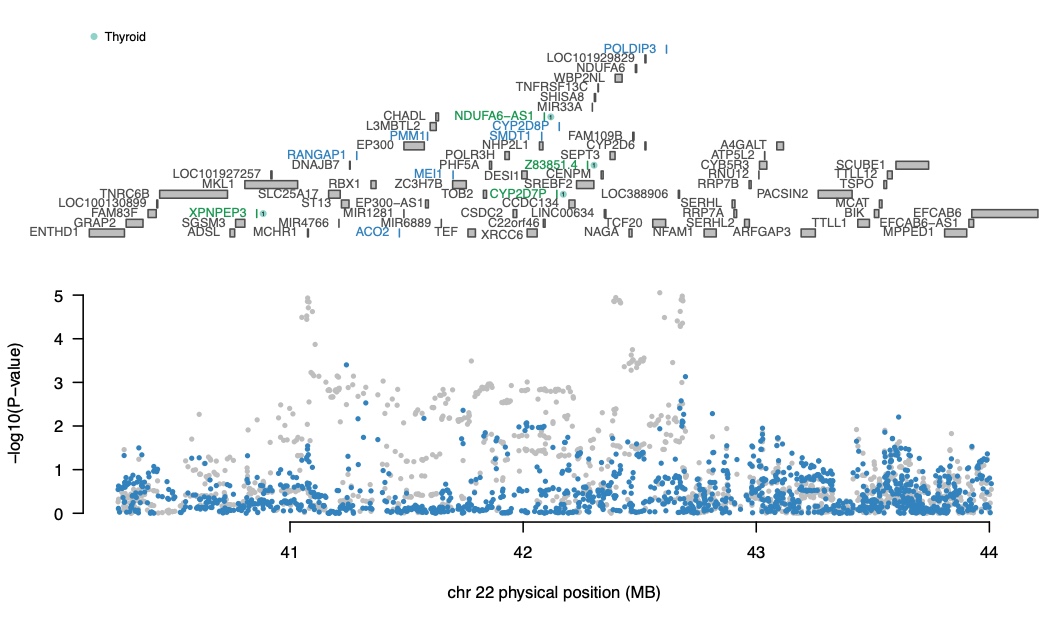 |
|  |
| 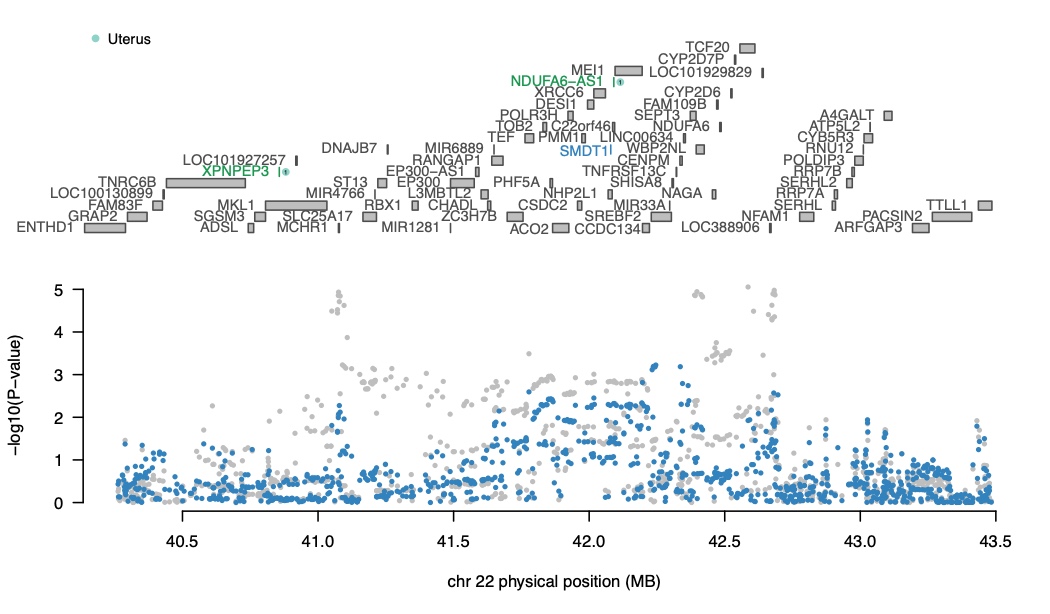 |
|  |
